# Assessment of serum pharmacokinetics and urinary excretion of albendazole and its metabolites in human volunteers

**DOI:** 10.1371/journal.pntd.0005945

**Published:** 2018-01-18

**Authors:** Laura Ceballos, Alejandro Krolewiecki, Marisa Juárez, Laura Moreno, Fabian Schaer, Luis I. Alvarez, Rubén Cimino, Judd Walson, Carlos E. Lanusse

**Affiliations:** 1 Laboratorio de Farmacología, Centro de Investigación Veterinaria de Tandil (CIVETAN), UNCPBA-CICPBA-CONICET, Facultad de Ciencias Veterinarias, UNCPBA, Campus Universitario, Tandil, Argentina; 2 Instituto de Investigaciones en Enfermedades Tropicales, Universidad Nacional de Salta, sede regional Orán. Alvarado, SRN, Oran, Salta, Argentina; 3 National History Museum, London, United Kingdom; 4 DeWorm3 Program, Departments of Global Health, Medicine, Pediatrics and Epidemiology, University of Washington, Seattle, United States of America; McGill University, CANADA

## Abstract

**Background:**

Soil Transmitted Helminth (STH) infections negatively impact physical and mental development in human populations. Current WHO guidelines recommend morbidity control of these infections through mass drug administration (MDA) using albendazole (ABZ) or mebendazole. Despite major reductions in STH associated morbidity globally, not all programs have demonstrated the expected impact on prevalence of parasite infections. These therapeutic failures may be related to poor programmatic coverage, suboptimal adherence or the exposure of parasites to sub-therapeutic drug concentrations. As part of the DeWorm3 project, we sought to characterize the serum disposition kinetics and pattern of urinary excretion of ABZ and its main metabolites ABZ sulphoxide (ABZSO) and ABZ sulphone (ABZSO_2_) in humans, and the assessment of the duration and optimal time point where ABZ and/or its metabolites can be measured in urine as an indirect assessment of an individual’s adherence to treatment.

**Methodology/Principal findings:**

Consecutive venous blood and urine samples were collected from eight (8) human volunteers up to 72 h post-ABZ oral administration. ABZ/metabolites were quantified by HPLC. The ABZSO metabolite was the main analyte recovered both in serum and urine. ABZSO Cmax in serum was 1.20 ± 0.44 μg/mL, reached at 4.75 h post-treatment. In urine, ABZSO Cmax was 3.24 ± 1.51 μg/mL reached at 6.50 h post-ABZ administration.

**Conclusion/Significance:**

Pharmacokinetic data obtained for ABZ metabolites in serum and urine, including the recovery of the ABZ sulphoxide derivative up to 72 h in both matrixes and the recovery of the amino-ABZ sulphone metabolite in urine samples, are suggesting the possibility of developing a urine based method to assess compliance to ABZ treatment. Such an assay may be useful to optimize ABZ use in human patients.

**Trial registration:**

ClinicalTrials.gov NCT03192449.

## Author summary

The soil-transmitted-helminths (STH) infections are produced by four species of parasites: *Ascaris lumbricoides*, *Trichuris trichiura*, and hookworm (*Necator americanus* and *Ancylostoma duodenale*). These parasites are transmitted by eggs present in human faeces, which contaminate the environment in areas where sanitation is poor. These diseases negatively impact health and development. Current STH control is based on mass drug administration (MDA) programs, mainly through the use of albendazole (ABZ) and mebendazole. However, although MDA programs have been shown to reduce the prevalence of STH infection and to control morbidity in school-age children, rapid reinfection is common among treated children and many programs fail to reach coverage targets. Optimizing methods to assess treatment coverage may allow programs to more effectively deliver MDA to populations requiring treatment. We conducted a controlled trial to evaluate the serum pharmacokinetic behaviour and urinary excretion of ABZ and its metabolites in human volunteers. The study focused also on to evaluate, by mean of non-invasively measures, ABZ treatment coverage and adherence. The measurement of ABZSO concentrations both in serum and urine may be useful methods to monitor adherence to ABZ treatment and serve as a more objective measurement of program coverage.

## Introduction

The neglected tropical diseases (NTDs) are a group of infections which affect the poorest regions of the world [1]. Among the NTDs, Soil-transmitted helminths (STH, the roundworm *Ascaris lumbricoides*, the whipworm *Trichuris trichiura* and the two hookworm species *Necator americanus* and *Ancylostoma duodenale*) are among the most prevalent parasites worldwide, disproportionately affecting human populations living without adequate water and sanitation [2]. It is estimated that more than 1.4 billion people are infected with at least one of the four STH species [3]. STH persist for years in the human gastrointestinal tract, negatively impacting nutritional status, individual productivity and physical and mental development [1, 4]. The World Health Organization (WHO) recommendations for control of STH include preventive chemotherapy through regular mass drug administration (MDA) for school age children and other at-risk groups living in endemic areas where prevalence of STH exceeds 20%. Currently, deworming is carried out with single oral dose of a benzimidazole (BZD) anthelmintic drug (albendazole (ABZ) or mebendazole (MBZ)) once or twice year depending on baseline prevalence [5].

Despite significant success in scaling up deworming programs and reducing associated morbidity, program coverage globally is still less than optimal [6]. In addition, some areas report apparent reductions in treatment efficacy following multiple rounds of MDA. Treatment failure may be related to poor programmatic coverage or adherence, reduced drug efficacy through suboptimal drug formulation, poor drug absorption or the development of resistance. It is critical to differentiate a lack of coverage or adherence to drug from issues related to drug quality or the development of drug resistance. Current coverage estimates rely on subjective reporting of coverage and adherence through an interview based coverage survey. Such instruments are prone to reporter and interviewer bias and may not adequately reflect true treatment coverage and/or adherence [7].

The development of improved tools to accurately determine treatment coverage and adherence may be important to enable programs to more accurately report true coverage of MDA and to respond to situations where treatment failure is suspected. In fact, validation of compliance is extremely important also for lymphatic filariasis elimination, and like with STH, there are currently no tools to use to measure it.

Accurate measurement and determination of coverage and adherence to MDA has become even more critical as the STH community begins to think beyond control towards the possibility of disease elimination. Although annual MDA may reduce the prevalence of STH infection and control morbidity in school-age children, reinfection following treatment is common and this strategy is likely to be insufficient to break STH transmission [6]. Recent mathematical modelling studies suggests that MDA might be sufficient to interrupt STH transmission if new strategies, including expanding MDA to adults and/or treating children more frequently, are used [8]. These models suggest that exceptionally high coverage and adherence will be needed to interrupt transmission through MDA alone.

The widespread use of BZD anthelmintics, particularly the methylcarbamate derivatives such as ABZ, is based on their high efficacy, low toxicity, and broad-spectrum of activity [9]. While there are limited data on the pharmacokinetics (PK) and urinary excretion of ABZ and its metabolites in humans, it is known is that after absorption, BZD compounds are extensively metabolized. Biotransformation takes place predominantly in the liver [10], although metabolic activity is apparent in extrahepatic tissues such as the lungs [11] and gastrointestinal (GI) tract [12]

Consequently, ABZ (the parent compound) is undetectable in the systemic circulation after administration [13–18]. ABZ is rapidly oxidized into its active metabolite ABZ-sulphoxide (ABZSO) and further liver oxidative and hydrolytic metabolism produces ABZ sulphone (ABZSO_2_) and albendazole amino sulphone (ABZ-SO2–NH_2_), respectively. ABZSO and ABZSO_2_ are the metabolites mainly found in the systemic circulation [15, 16]. ABZSO appears to be the main metabolite that is recoverable in human plasma [13, 19–21] and in urine [22].

As part of the DeWorm3 project, a large series of community cluster randomized trials to determine the feasibility of interrupting STH transmission, we sought 1) to develop and validate an analytical method to quantify concentrations of ABZ and its metabolites, ABZSO and ABZSO_2_, in human serum and urine samples, 2) to conduct a study of the serum pharmacokinetics and urinary excretion of ABZ and its metabolites in human volunteers, and 3) to determine the optimal and the longest time period after treatment when either ABZ and/or its metabolites can be measured in urine as an indirect assessment of an individual’s adherence to treatment.

## Materials and methods

### Ethical aspects

The experimental protocol and Informed Consent Form (ICF) were approved by the Bioethics Committee at the Colegio Médico de Salta (Argentina).

All the volunteers provided written consent for their participation in the study before any study procedures commenced. Volunteers received a small stipend for their collaboration. The trial is registered at clinicaltrials.gov (REF NCT03192449).

### Study population

Adults age between 18 and 45 years and weighting up to 75 Kg with no known chronic medical conditions and a normal physical exam were recruited for participation. Female volunteers had to use reliable contraceptive measures to be eligible to participate. Exclusion criteria were: Intake of ABZ or other BZD drugs within the last 30 days; malabsorption or other syndromes that could compromise the tolerability or absorption of ABZ; history of hypersensitivity or intolerance to ABZ or its inactive ingredients; acute clinical condition; pregnancy or breast feeding.

### Chemicals

Pure reference standards of ABZ, ABZSO, ABZSO_2_ and oxibendazole (OBZ) (99% purity) were purchased from Sigma–Aldrich (St. Louis, MO, USA). The HPLC grade solvents acetonitrile and methanol were from Baker, Mallinckrodt (Baker, Phillipsburg, USA). Ethyl acetate was from Anedra (BA, Argentina). Water was distilled and deionized using a water purification system (Simplicity, Millipore, São Paulo, Brazil). ABZ tabs (400mg) administered to human volunteers was provided by GlaxoSmithKline.

### Experimental design

Eight (8) healthy volunteers (four male and four female, 18–40 years of age; body weight between 53 and 75 kg) participated in the trial. The treatment and sampling phases of the study were conducted at the Laboratory of the “Instituto de Investigaciones en Enfermedades Tropicales” at the Oran branch of the Universidad Nacional de Salta, Argentina.

Prior to ABZ treatment, all subjects were provided with a standard meal (fat content 40 g) and baseline blood (5 mL) and urine (20 mL) samples were obtained (sampling time = 0). After 15 min a single postprandial (fat content 40 g) oral dose of ABZ (400 mg) was administered and consecutive venous blood samples were collected by a registered nurse at 2, 4, 8, 12, 24, 36, 48 and 72 h post-treatment (p.t.). Blood was collected into tubes and centrifuged immediately. Serum samples were stored at −70°C until assayed. Urine samples were collected at the following specific times: 4, 8, 12, 24, 36, 48 and 72 h. Samples were stored at -20°C until HPLC analysis of ABZ/metabolites.

### Chromatographic analysis of ABZ/metabolites in collected samples

#### Serum samples extraction

Serum samples (1 mL) were placed into plastic tubes (5 mL). The samples were spiked with 40 μL of OBZ-IS (25 μg/mL) and the molecules to be assayed (ABZ, ABZSO, ABZSO_2_) in the validation procedure. Drug molecules were extracted from serum by a solid phase extraction (SPE) procedure. SPE was made using C_18_ cartridges (Strata, RP-18 100 mg, Phenomenex, CA, USA) previously conditioned with 1 mL of methanol, followed by 1 mL of water. The sample was applied and then sequentially washed with 3 mL of HPLC water, dried with air for 5 min and eluted with 2 mL of methanol. The elute was evaporated to dryness under a gentle stream of nitrogen at 56°C in a water bath (Zymark TurboVap LV evaporator. American Laboratory Trading, Inc. Lyme 06333 CT USA). The dry residue was dissolved in 250 μL of mobile phase (acetonitrile:water, 27:73). An aliquot (50 μL) of this solution was injected in the chromatographic system.

#### Urine samples extraction

Urine samples (3 mL) were placed into plastic tubes (10 mL). The samples were spiked with 60μL of OBZ-IS (25 μg/mL) and the molecules to be assayed (ABZ, ABZSO, ABZSO_2_) in the validation procedure. Analytes were extracted by addition of 3 mL of ethyl acetate. After shaking (vortex 50 min), and centrifugation (2300 g, 15 min, 4°C), the clear supernatant (ethyl acetate phase) was transferred to a 10 mL glass tube. The samples were extracted again with 3 mL of ethyl acetate as described above. The total supernatant (6 mL approx.) was evaporated to dryness under a gentle stream of dry nitrogen at 56°C in a water bath. The dry residue was dissolved in 250 μL of mobile phase (acetonitrile:water, 27:73) and vigorously shaken (15 min). Finally, the samples were transferred to an eppendorf vial and centrifuged (16200 g, 20 min, 4°C) to inject 50 μL of this solution directly in the chromatographic system.

### HPLC system and chromatographic conditions

Experimental and fortified serum and urine samples were analyzed for ABZ, ABZSO and ABZSO_2_ by HPLC. After extraction, fifty (50) μL of sample was injected into a Shimadzu Chromatography System (Shimadzu Corporation, Kyoto, Japan). The equipment is composed for a LC-20AT quaternary pump, an automatic sample injector (SIL-10AF), an ultraviolet visible spectophotometric detector (UV) (SPD-20A) set at a wavelength of 292 nm, a column oven (CTO-10AS vp) set at 30°C, and a CBM-20A data integrator. Data and chromatograms were collected and analyzed using the Class LC10 software (SPD-10A, Shimadzu Corporation, Kyoto, Japan). A C_18_ reversed-phase column (Gemini, Phenomenex, USA) of 250 x 4.6 mm with 5 μm particle size was used for separation. Elution from the stationary phase was carried out at a flow rate of 1.2 mL/min using acetonitrile and ammonium acetate buffer (0.025 M, pH 6.6) as the mobile phase that was pumped with variable gradient (acetonitrile: ammonium acetate buffer) during the run (16 min). The gradient changed from 27:73 to 50:50 in 5 min, then maintained for 7 min and modified to 27:73 in 1 min, in which was maintained during 4 min. The compounds were identified using the retention times of 99% pure reference standards.

### Validation of the analytical methodology

Before starting the measurement of ABZ, ABZSO and ABZSO_2_ concentrations in serum and urine samples, a complete validation of the analytical methodologies was performed. The details of the validation of the analytical methodology (both in serum and urine) and their results are explained as supplementary information.

### Pharmacokinetic analysis of the serum and urine concentration data

The pharmacokinetic analysis of the serum and urine concentrations obtained after ABZ single oral administration (400 mg) was performed using the program PK Solution 2.0 (Summit Research Services, Ashland, USA), and pharmacokinetic analysis was performed using non compartmental (area) and compartmental (exponential terms) methods without presuming any specific compartmental model. The peak concentration (C_max_) and time to peak concentration (T_max_) were displayed from the plotted concentration-time curve of each analyte. The formation half-life (T_½for_) and the elimination half-life (T_½el_) were calculated as ln2/k_abs_ and ln2/λ_el,_ respectively, where k_for_ represents the first order formation rate constant and λ_el_ is the elimination rate constant. The area under the concentration time-curve (AUC) was calculated by means the trapezoidal rule [23] up to 72 h, and further extrapolated to infinity by dividing the last experimental concentration by the terminal slope (β). Statistical moment theory was applied to calculate the mean residence time (MRT) in serum as follows: MRT = AUMC/AUC; where AUC is defined previously and AUMC is the area under the curve of the product of time and the serum drug concentration vs. time from zero to infinity [24].

### Analysis of the data

Data are expressed as arithmetic mean ± standard deviations (SD). Correlation between individual serum/urine human concentrations was performed by parametric analysis (Pearson r, r^2^).

## Results

Results of validation of the analytical methodology are detailed as a supporting S1 Text (Tables A-M in S1 Text and S1, S2 and S3 Figs. TIFF)

### Pharmacokinetic analysis of the serum and urine concentration data

The validated method was successfully applied to quantify ABZ/metabolites in both serum and urine samples from humans treated with ABZ (400mg).

**Fig 1:**
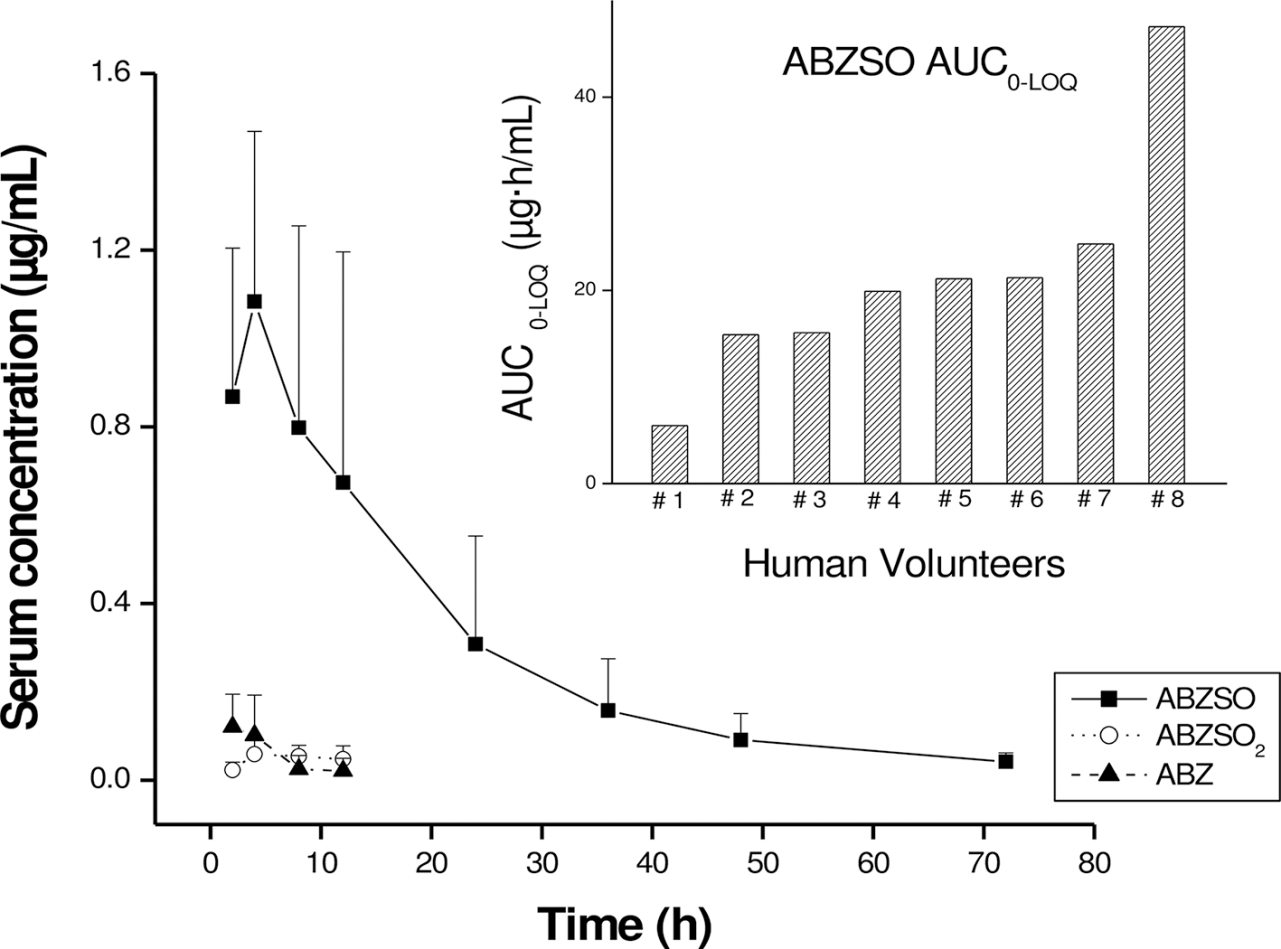
Serum concentration profiles for ABZ, ABZSO and ABZSO_2_. Comparative (mean ± SD) serum concentration profiles of albendazole sulphoxide (ABZSO), albendazole sulphone (ABZSO_2_) and albendazole (ABZ) obtained after ABZ administration as a single oral dose (400 mg) to human volunteers. The insert shows the individual area under the concentration vs. time (AUC _0-LOQ_) of ABZSO as an indicator of systemic drug availability.

After ABZ administration, only trace amounts of ABZ parent drug were detected in serum between 2 and 8 h p.t.. ABZ was rapidly metabolized, with ABZSO and ABZSO_2_ measured in the bloodstream at the first sampling time (2 h p.t.). The pharmacologically active ABZSO metabolite was the analyte recovered at the highest concentrations which rapidly increased to reach its peak concentration (C_max_ = 1.20 ± 0.44 μg/mL) at 4.75 h (T_max_) p.t.. The systemic drug exposure, estimated as the ABZSO AUC _0-LOQ_ value, was 21.4 ± 1.19 μg·h/mL. This analyte was measured in the bloodstream up to 72 h after ABZ oral administration in seven volunteers (volunteer 2–7). In serum sample collected from volunteer #1, ABZSO was detected up to 24 h p.t.. The mean serum concentration profiles vs time for ABZ, ABZSO and ABZSO_2_ and the individual AUC_0-LOQ_ value for ABZSO obtained after ABZ administration are shown in Fig 1. Low (and erratic) concentrations of the inactive ABZSO_2_ metabolite were quantified between 4 and 12 h p.t. in some of the treated volunteers. The pharmacokinetic analysis of ABZ and its ABZSO_2_ in serum was not performed, since most of the measured concentration values were under the LOQ. The complete pharmacokinetic analysis, including individual and mean (±SD) serum pharmacokinetic parameters obtained for ABZSO are shown in Table 1.

**Table 1:** ABZSO serum pharmacokinetic parameters.

Pharmacokinetic parameters	Volunteers #									
									
	1	2	3	4	5	6	7	8	Mean	SD
**T**_**1/2 for**_ **(h)**	ND	1.72	0.85	0.91	0.69	0.64	1.06	2.40	**0.53**	0.65
**C**_**max**_ **(μg/mL)**	0.65	0.66	0.94	1.17	1.37	1.24	1.63	1.90	**1.20**	0.44
**T**_**max**_ **(h)**	2	4	4	4	4	4	4	12	**4.75**	3.01
**AUC**_**(0-LOQ)**_ **(μg·h/mL)**	6.00	15.4	15.6	19.9	21.2	21.3	24.8	47.3	**21.4**	11.9
**AUC**_**(0-∞)**_ **(μg·h/mL)**	6.20	16.8	16.2	20.6	22.2	23.7	25.6	48.2	**22.4**	12.0
**T**_**1/2 el**_ **(h)**	12.7	17	13.9	13.8	13.5	18.0	13.1	11.6	**14.20**	2.19
**MRT (h)**	14.9	24.8	18.1	20.5	19.5	26.9	18.6	20.5	**20.4**	3.80

Serum pharmacokinetic parameters (mean ± SD) for albendazole sulphoxide (ABZSO) obtained after its administration as a single oral dose (400 mg) to human volunteers.

T_1/2 for_: Formation half-life; C_max_: Peak serum concentration; T_max_: Time to peak serum concentration; AUC_(0-LOQ)_:Area under the concentration vs. time curve from time 0 to the limit of quantification; AUC_(0-∞)_: Area under the concentration vs. time curve extrapolated to infinity; T_½el_: Elimination half-life; MRT: Mean residence time; SD: Standard deviation. ND = not determined.

**Fig 2:**
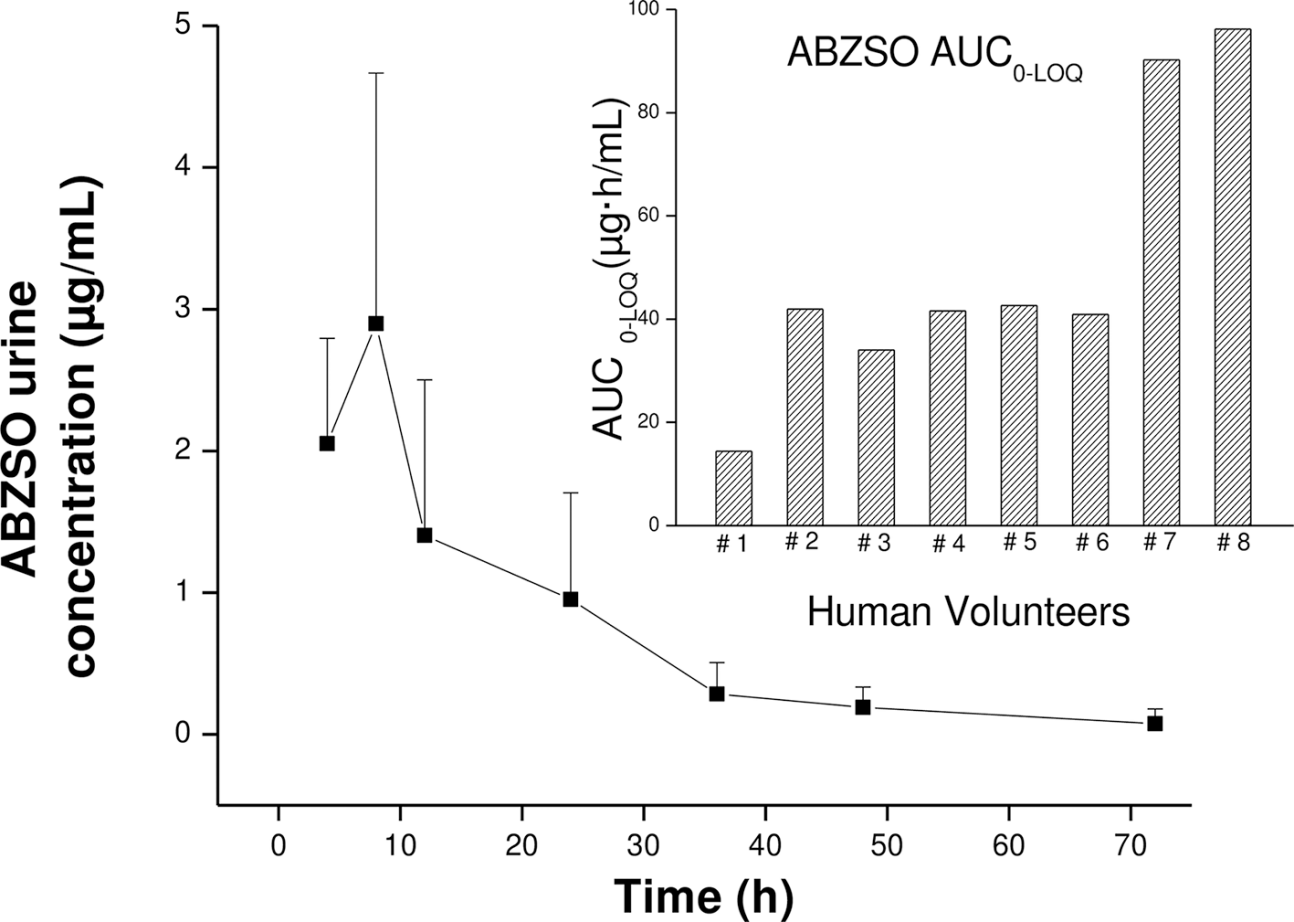
Urine concentration profile for ABZSO. Albendazole sulphoxide (ABZSO) mean (±SD) urine concentration vs. time profiles obtained after albendazole (ABZ) administration as a single oral dose (400 mg, GlaxoSmithKline) to human volunteers. The insert shows the Individual area under the concentration vs. time (AUC_0-LOQ_) of ABZSO after ABZ administration.

**Fig 3:**
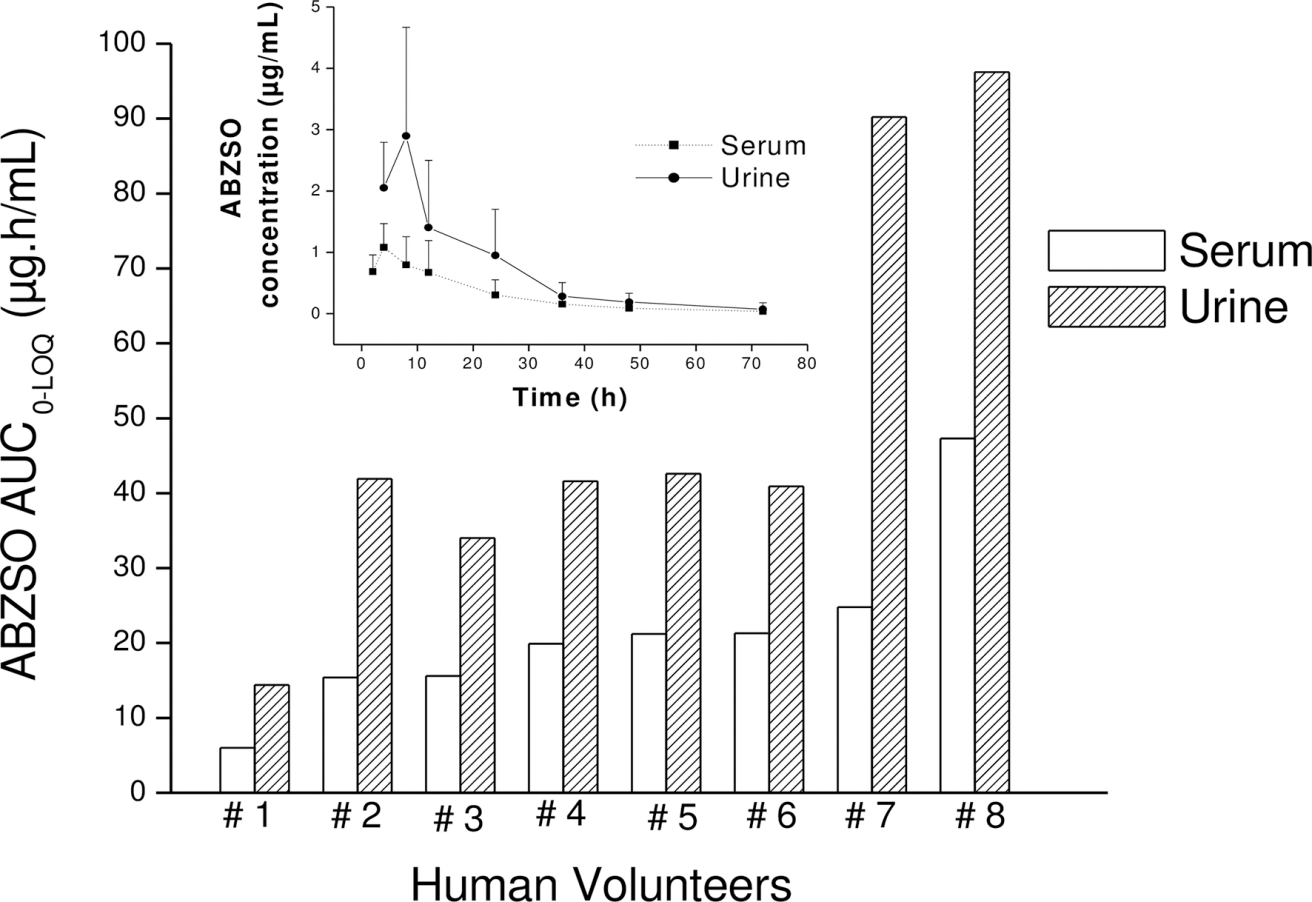
ABZSO systemic exposure in serum and urine. Comparative albendazole sulphoxide (ABZSO) individual serum and urine area under concentration vs time curve (AUC_0-LOQ_) obtained from albendazole-treated human volunteers (400 mg, GlaxoSmithKline). The insert shows ABZSO concentrations (mean ± SD) both in serum and urine samples from treated volunteers.

The ABZSO metabolite was rapidly excreted in urine following the ABZ treatment and was the main ABZ metabolite recovered between 4 h (first sampling time) and 72 h p.t.. ABZSO peak urine concentration (3.24 ± 1.51 μg/mL) was reached at 6.50 h p.t. (T_max_). The ABZSO urinary exposure, (estimated as the AUC) was 50.2 μg·h/mL (Table 2) which resulted higher (2.3 fold) than that measured in serum. Low concentrations of ABZSO_2_ were quantified in urine between 4 and 8 h p.t., mostly under the LOQ which precluded any pharmacokinetic analysis. ABZ concentrations in urine were under the limit of detection at all sampling times. The mean urine concentration profile vs. time for ABZSO and its individual AUC_0-LOQ_ value is illustrated in Fig 2. The individual and mean (±SD) pharmacokinetic parameters describing the urinary excretion of ABZSO are shown in Table 2. Fig 3 shows the individual ABZSO AUC value both in serum and urine samples obtained from treated volunteers, as well as the comparative ABZSO concentrations in both matrixes up to 72 h p.t..

**Table 2:** Parameters describing the urinary excretion of ABZSO.

Pharmacokinetic Parameter	Volunteers #									
									
	1	2	3	4	5	6	7	8	Mean	SD
**C**_**max**_ **(μg/mL)**	1.40	3.38	2.46	2.40	2.30	3.00	5.85	5.12	**3.24**	1.51
**T**_**max**_ **(h)**	8	8	8	4	4	4	8	8	**6.50**	2.07
**AUC**_**(0-LOQ)**_**(μg·h/mL)**	14.4	41.9	34.0	41.6	42.6	40.9	90.2	96.2	**50.2**	28.2
**AUC**_**(0-∞)**_**(μg·h/mL)**	14.4	42.6	34.1	41.3	44.2	42.1	90.5	102.7	**51.5**	29.6

Urine pharmacokinetic parameters (mean ± SD) for albendazole sulphoxide (ABZSO) obtained after albendazole (ABZ) administration as a single oral dose (400 mg) to human volunteers.

C_max_: Peak concentration; T_max_: Time to peak concentration; AUC_(0-LOQ)_: Area under the concentration vs. time curve from time 0 to the limit of quantification; AUC_(0-∞)_: Area under the concentration vs. time curve extrapolated to infinity. SD: standard deviation

**Fig 4:**
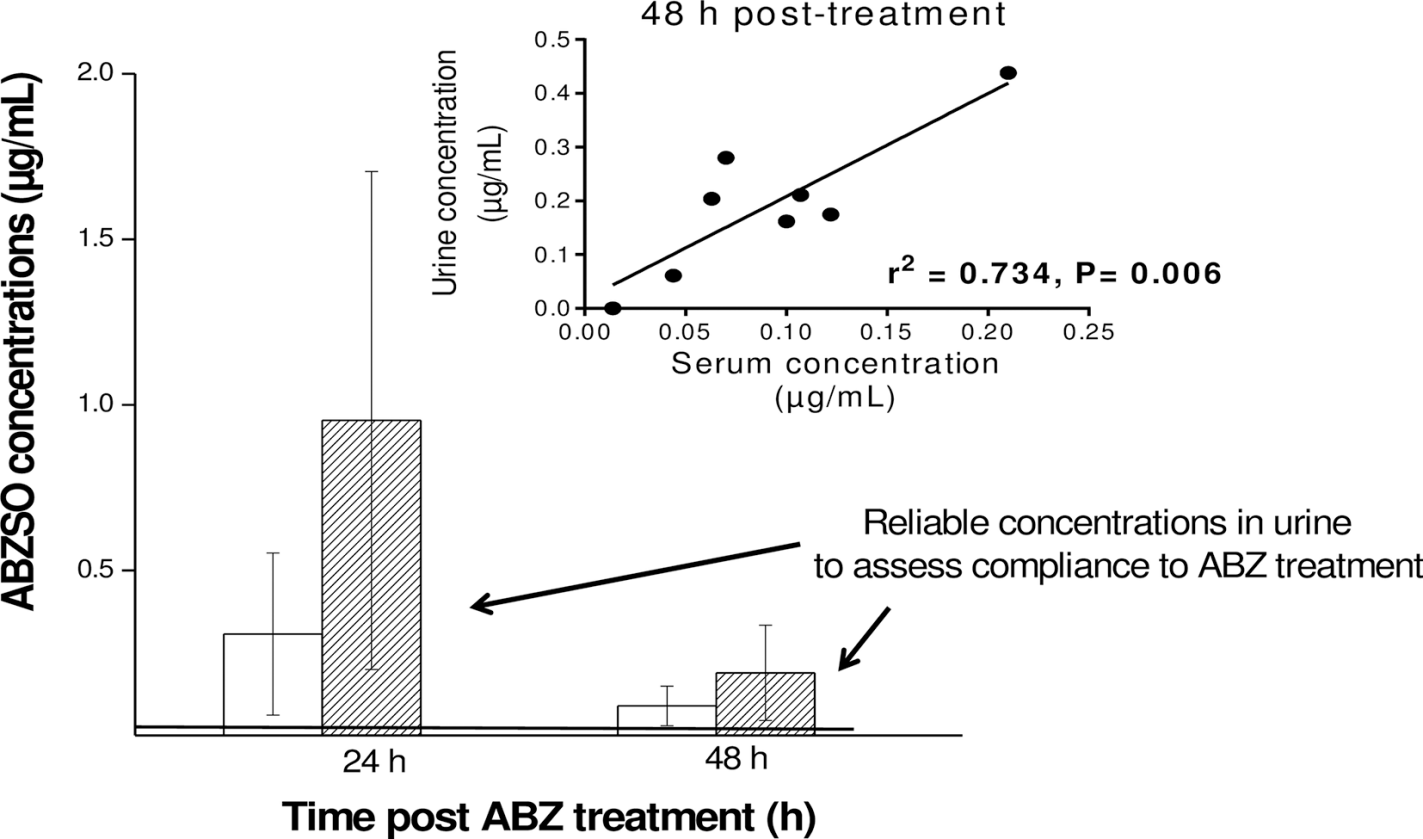
Comparative ABZSO serum and urine concentrations. Comparative albendazole sulphoxide (ABZSO) serum and urine concentrations (mean ± SD) obtained from human volunteers at 24 and 48 h post albendazole (ABZ)-treatment (400 mg, GlaxoSmithKline). The straight-line represents the limit of quantification (LOQ) value determined for the used methodology. The insert shows correlation between individual ABZSO concentration in serum and urine samples at 48 h post ABZ treatment. The straight-line represents the best-fit line obtained by linear regression analysis.

High correlation was obtained between individual ABZSO concentrations in serum and urine either at 24 or 48 h after ABZ administration. The Pearson correlation coefficient values were 0.674 (24h) and 0.734 (48h) (P value 0.01 (24h) and 0.006 (48 h)). Fig 4 shows the mean ABZSO concentrations obtained in serum and urine samples at 24 and 48 h post ABZ-treatment and the correlation between individual ABZSO concentration in serum and urine samples (48 h p.t.).

From the analysis of the urine samples from ABZ-treated volunteers, another relevant chromatographic peak was observed in addition to the ABZSO metabolite peak (S3 Fig TIFF). This chromatographic peak was chemically identified as an oxidative amino-ABZ sulphone derivative. The mean retention time was 4.11 min under the previously described chromatographic conditions and it was detected between 4 and 72 h p.t. Although this metabolite was not included in the validation of the analytical methodology, its presence was checked by direct injections of known quantities of an amino-ABZSO_2_ reference standard (99% purity) solutions in mobile phase.

## Discussion

A major factor contributing to the success of STH control programs is the true coverage and adherence achieved in MDA programs [25]. Here we report promising preliminary data suggesting that it may be possible to develop simple tools to measure metabolites of ABZ in urine to assess treatment coverage/adherence.

Optimizing methods to determine coverage and adherence in treated populations offers the opportunity to accurately assess the performance of MDA programs in reaching and treating targeted individuals. In addition, these tools allow programs to modify activities based on a clearer understanding of factors contributing to less than expected STH prevalence and/or intensity reductions. Finally, as concerns emerge regarding the potential for the emergence of drug resistance to commonly used anthelmintics; accurate determination of coverage and adherence allows programs to evaluate whether potential treatment failure is due to the possible emergence of resistance mutations or is simply a result of lower than expected treatment coverage or adherence.

In the present study, the serum pharmacokinetic of ABZ was tested up to 72 h post-administration of ABZ. The rapid appearance of ABZSO and ABZSO_2_ in serum and fast removal of the parent drug, confirm the first pass liver, GI and lungs microsomal oxidation of ABZ previously proposed [10–13]. ABZSO was the main analyte detected in serum samples and was measured up to the last treatment point at 72 hours p.t.. The low concentrations measured for ABZSO_2_ metabolite, precluded any pharmacokinetic analysis. The ABZSO pharmacokinetic parameters C_max_ (1.20 ± 0.44 μg/mL) and AUC (21.4 ± 11.9 μg.h/mL) obtained here are consistent with previously reported data [19, 20, 26]. The high variations in ABZSO serum concentrations among subjects, observed specially in volunteers #1 (lowest serum exposure) and # 8 (highest serum exposure) are in concordance with previous reported studies [26, 27] and may be explained by intrinsic differences among the volunteers, and may be related to individual differences in drug absorption.

The overall serum PK parameters for ABZSO observed in the current work are different from those previously reported [19, 21], as ABZSO was quantified in higher concentrations and for longer period. The subjects involved in the current work were dosed post-prandially after a 40g fat meal. The administration of ABZ with food increases the permanence of drug formulation at the stomach, maximizing its dissolution and systemic exposure. However, this situation will not necessarily match with the real situation in the field, in which drug exposure levels after ABZ administration in “fasted” patients would be likely much lower.

BZD methylcarbamates are formulated as tablets and show only limited GI absorption due to their poor solubility in water. In humans, the stomach plays a critical role in drug dissolution, since the low pH of the stomach fluid facilitates BZD water dissolution, which is the rate-limiting step in the systemic availability of the active drug/metabolites [28]. The plasma concentration profiles of ABZSO reflect the amount of dissolved drug at GI level and the overall drug exposure within the GI tract [29].

We also report here for the first time a complete urinary excretion profile of ABZ/metabolites in humans. ABZSO was the main analyte recovered in urine samples, in agreement with limited data found in the literature [22, 30]. The urinary excretion of ABZSO was very efficient and the analyte could be measured from the first time point p.t. (4 h) at high concentrations (2.52 ± 0.74 μg/mL) and remained detectable up to 72 h p.t.. Relevant concentrations were detected at 24 (0.95 ± 0.75 μg/mL) and 48 h (0.19 ± 0.14 μg/mL) with all measures over the LOQ obtained for this metabolite. ABZSO was quantified at three days p.t. in seven of eight volunteers, at concentrations close to the LOQ (0.08 ± 0.10 μg/mL). In addition, the ABZSO Cmax in urine resulted 2.8 times higher than measured in the bloodstream, and the drug exposure, estimated as the ABZSO AUC value resulted 2.3 fold higher compared to that measured in serum. These results are promising for the potential to develop a field ready tool to evaluate adherence to treatment using non-invasively obtained biological samples such as urine, collected up to two days after ABZ administration. This “optimal” time refers to that in which the ABZSO concentration can be accurate (above the limit of quantification) measured with the validated methodology, and it should also be a practical sampling period for the staff involved in the implementation of control programs. It is important to highlight that the use of urinary metabolite detection as a useful way of monitoring for treatment compliance may depend on the quality of the pharmaceutical product. Drug concentration achieved in bloodstream by drug orally administered depends on the chemical properties of the drug and the pharmaceutical preparations in which the active compound is formulated. The current work has been performed with a specific product (ABZ tabs, GlaxoSmithKline) currently used in the MDAs programs. However, there are a wide range of other brands available on local markets of STH endemic countries, which are often more accessible to the local people, but for which the quality remain poorly explored. For instance, the use of this methodology to evaluate treatment compliance can be recommended after administration of a high quality ABZ formulation. Finally, a high positive correlation was observed between ABZSO concentrations in serum and urine in human volunteer at 24 and 48 h after ABZ administration, which could be useful to indirectly estimate ABZSO concentrations in serum, predicting any difference in drug absorption among individuals.

As an additional contribution of the present trial, the amino-ABZSO_2_ metabolite was also recovered (between 4 and 72 h) in urine samples from treated human volunteers, which would be a novel approach/alternative to monitor treatment adherence. Amino-ABZSO_2_ has been identified in the bloodstream in cattle, pigs, sheep and humans [18, 31, 32], and in urine from sheep [31, 33] after ABZ administration. This may represent a novel metabolite that can also be measured by the validated methodology without any change to monitor treatment adherence.

The analytical methodology developed for this project was simple, well-defined, reproducible, precise, accurate and easy to perform. That validated methodology was successfully applied to quantify ABZ and its metabolites, ABZSO and ABZSO_2_, in both serum and urine samples from treated human volunteers, and could be also used to quantify amino-ABZSO_2_.

The pharmacokinetic data obtained for ABZ metabolites in serum and urine, the recovery of the ABZ sulphoxide derivative up to 72 h in both matrixes and the recovery of the amino- ABZSO_2_ in urine samples, are scientifically solid contributions to optimize ABZ use in MDA interventions through the development of a tool to accurately assess coverage and adherence to ABZ treatment and obtain reliable coverage rates.

## Supporting information

S1 Text Supporting tables. Table Aa: Linearity of the detector response after injections of serum samples fortified with albendazole sulphoxide (ABZSO) from 0.025 to 2 μg/mL.
Table Ab: Linearity of the detector response after injections of serum samples fortified with albendazole sulphone (ABZSO_2_) from 0.025 to 2 µg/mL.
Table Ac: Linearity of the detector response after injections of serum samples fortified with albendazole (ABZ) from 0.025 to 2 μg/mL.
Table Ba: Linearity of the detector response after injections of urine samples fortified with albendazole sulphoxide (ABZSO) from 0.025 to 5 μg/mL.
Table Bb: Linearity of the detector response after injections of urine samples fortified with albendazole sulphone (ABZSO_2_) from 0.025 to 1 μg/mL.
Table Bc: Linearity of the detector response after injections of urine samples fortified with albendazole (ABZ) from 0.025 to 1 μg/mL.
Table Ca: Absolute recovery (%) of the method to quantify albendazole sulphoxide (ABZSO) by HPLC with UV detection in humans serum (n = 6).
Table Cb: Absolute recovery (%) of the method to quantify albendazole sulphone (ABZSO_2_) by HPLC with UV detection in human serum (n = 6).
Table Cc: Absolute recovery (%) of the method to quantify albendazole (ABZ) by HPLC with UV detection in human serum (n = 6).
Table Da: Absolute recovery (%) of the method to quantify albendazole sulphoxide (ABZSO) by HPLC with UV detection in human urine (n = 6).
Table Db: Absolute recovery (%) of the method to quantify albendazole sulphone (ABZSO_2_) by HPLC with UV detection in human urine (n = 6).
Table Dc: Absolute recovery (%) of the method to quantify albendazole (ABZ) by HPLC with UV detection in human urine (n = 6).
Table Ea: Interday precision of the method to quantify albendazole sulphoxide (ABZSO) by HPLC with UV detection in human serum (n = 6).
Table Eb: Interday precision of the method to quantify albendazole sulphone (ABZSO_2_) by HPLC with UV detection in human urine (n = 6).
Table Ec: Interday precision of the method to quantify albendazole (ABZ) by HPLC with UV detection in human serum (n = 6).
Table Fa: Interday precision of the method to quantify albendazole sulphoxide (ABZSO) by HPLC with UV detection in human urine (n = 6).
Table Fb: Interday precision of the method to quantify albendazole sulphone (ABZSO_2_) by HPLC with UV detection in human urine (n = 6).
Table Fc: Interday precision of the method to quantify albendazole (ABZ) by HPLC with UV detection in human serum (n = 6).
Table Ga: Interday accuracy of the method to quantify albendazole sulphoxide (ABZSO) by HPLC with UV detection in human serum (n = 6).
Table Gb: Interday accuracy of the method to quantify albendazole sulphone (ABZSO_2_) by HPLC with UV detection in human serum (n = 6).
Table Gc: Interday accuracy of the method to quantify albendazole (ABZ) by HPLC with UV detection in human serum (n = 6).
Table Ha: Interday accuracy of the method to quantify albendazole sulphoxide (ABZSO) by HPLC with UV detection in human urine (n = 6).
Table Hb: Interday accuracy of the method to quantify albendazole sulphone (ABZSO_2_) by HPLC with UV detection in human urine (n = 6).
Table Hc: Interday accuracy of the method to quantify albendazole (ABZ) by HPLC with UV detection in human urine (n = 6).
Table Ia: Albendazole sulphoxide (ABZSO) stability in human serum after freezing (-18 ºC) and after three freeze/thaw cycles. The parameter was expressed as coefficient of variation (%) (CV).
Table Ib: Albendazole sulphone (ABZSO_2_) stability in human serum after freezing (-18°C) and after three freeze/thaw cycles. The parameter was expressed as coefficient of variation (%) (CV).
Table Ic: Albendazole (ABZ) stability in human serum after freezing (-18°C) and after three freeze/thaw cycles. The parameter was expressed as coefficient of variation (%) (CV).
Table Ja: Albendazole sulphoxide (ABZSO) stability in human urine after freezing (-18°C) and after three freeze/thaw cycles. The parameter was expressed as coefficient of variation (%) (CV).
Table Jb: Albendazole sulphone (ABZSO_2_) stability in human urine after freezing (-18°C) and after three freeze/thaw cycles. The parameter was expressed as coefficient of variation (%) (CV).
Table Jc: Albendazole (ABZ) stability in human urine after freezing (-18°C) and after three freeze/thaw cycles. The parameter was expressed as coefficient of variation (%) (CV).
Table Ka: Limit of detection (LOD) determination for albendazole sulphoxide (ABZSO) in human serum (n = 6).
Table Kb: Limit of detection (LOD) determination for albendazole sulphone (ABZSO_2_) in human serum (n = 6).
Table Kc: Limit of detection (LOD) determination for albendazole (ABZ) in human serum (n = 6).
Table La: Limit of detection (LOD) determination for albendazole sulphoxide (ABZSO) in human urine (n = 9).
Table Lb: Limit of detection (LOD) determination for albendazole sulphone (ABZSO_2_) in human urine (n = 9).
Table Lc: Limit of detection (LOD) determination for albendazole (ABZ) in human urine (n = 9).
Table Ma: Albendazole sulphoxide (ABZSO) limit of quantification (LOQ) determined in human serum. Recovery, accuracy and precision in the LOQ are reported.
Table Mb: Albendazole sulphone (ABZSO_2_) limit of quantification (LOQ) determined in human serum. Recovery, accuracy and precision in the LOQ are reported.
Table Mc: Albendazole (ABZ) limit of quantification (LOQ) determined in human serum. Recovery, accuracy and precision in the LOQ are reported.
Table Na: Albendazole sulphoxide (ABZSO) limit of quantification (LOQ) determined in human urine. Recovery, accuracy and precision in the LOQ are reported.
Table Nb: Albendazole sulphone (ABZSO_2_) limit of quantification (LOQ) determined in human urine. Recovery, accuracy and precision in the LOQ are reported.
Table Nc: Albendazole (ABZ) limit of quantification (LOQ) determined in human urine. Recovery, accuracy and precision in the LOQ are reported.
(DOCX)

S1 Fig Chromatograms obtained from (A) drug-free *serum* sample spiked with oxibendazole (c) (as internal standard); (B) Serum sample spiked with albendazole sulphoxide; (a) (4.3 min), albendazole sulphone (b) (6.7 min), oxibendazole (c) and albendazole (d) (11.7 min).
(TIF)

S2 Fig Chromatograms obtained from (A) drug-free urine sample spiked with oxibendazole (c) (as internal standard); (B) Urine sample spiked with albendazole sulphoxide; (a) (4.3 min), albendazole sulphone (b) (6.7 min), oxibendazole (c) and albendazole (d) (11.7 min).
(TIF)

S3 Fig Shows the chromatograms corresponding to the amino-ABZSO_2_ metabolite spiked in mobile phase samples (A), and to an experimental urine sample (B), albendazole amino sulphone (a) (4.11 min); albendazole sulphoxide(b) (4.3 min); oxibendazole (c) (6.7 min).
(TIF)
