# Supplementary material for: Assessment of serum pharmacokinetics and urinary excretion of albendazole and its metabolites in human volunteers
Source: PLoS Negl Trop Dis. 2018 Jan 18;12(1):e0005945. doi: 10.1371/journal.pntd.0005945 (PMC5773000; doi:10.1371/journal.pntd.0005945)
Supplement: S1 Text — Table Aa: Linearity of the detector response after injections of serum samples fortified with albendazole sulphoxide (ABZSO) from 0.025 to 2 μg/mL. Table Ab: Linearity of the detector response after injections of serum samples fortified with albendazole sulphone (ABZSO2) from 0.025 to 2 µg/mL. Table Ac: Linearity of the detector response after injections of serum samples fortified with albendazole (ABZ) from 0.025 to 2 μg/mL. Table Ba: Linearity of the detector response after injections of urine samples fortified with albendazole sulphoxide (ABZSO) from 0.025 to 5 μg/mL. Table Bb: Linearity of the detector response after injections of urine samples fortified with albendazole sulphone (ABZSO2) from 0.025 to 1 μg/mL. Table Bc: Linearity of the detector response after injections of urine samples fortified with albendazole (ABZ) from 0.025 to 1 μg/mL. Table Ca: Absolute recovery (%) of the method to quantify albendazole sulphoxide (ABZSO) by HPLC with UV detection in humans serum (n = 6). Table Cb: Absolute recovery (%) of the method to quantify albendazole sulphone (ABZSO2) by HPLC with UV detection in human serum (n = 6). Table Cc: Absolute recovery (%) of the method to quantify albendazole (ABZ) by HPLC with UV detection in human serum (n = 6). Table Da: Absolute recovery (%) of the method to quantify albendazole sulphoxide (ABZSO) by HPLC with UV detection in human urine (n = 6). Table Db: Absolute recovery (%) of the method to quantify albendazole sulphone (ABZSO2) by HPLC with UV detection in human urine (n = 6). Table Dc: Absolute recovery (%) of the method to quantify albendazole (ABZ) by HPLC with UV detection in human urine (n = 6). Table Ea: Interday precision of the method to quantify albendazole sulphoxide (ABZSO) by HPLC with UV detection in human serum (n = 6). Table Eb: Interday precision of the method to quantify albendazole sulphone (ABZSO2) by HPLC with UV detection in human urine (n = 6). Table Ec: Interday precision of the method to quantify albenda [file pntd.0005945.s001.docx]

**VALIDATION OF THE ANALYTICAL METHODOLOGY**

Stock and working solutions of a mix of the pure references standards (ABZ + ABZSO + ABZSO_2_) in methanol were prepared. To validate the methodology for the serum and urine kinetics studies, the following parameters were determined: *Selectivity, Linearity, Precision, Accuracy, Limit of Detection, Limit of Quantification and Stability.*

Before starting the measurement of ABZ and/or metabolite concentrations in serum and urine samples, A full validation of the analytical procedures for the extraction and quantification of each molecule (ABZ, ABZSO and ABZSO_2_) in each matrix (serum and urine) was performed following internationally accepted parameters (Ich Harmonised Tripartite Guideline, 2014) for chromatographic methodologies, including assessment of absolute analyte recoveries, linearity, precision and accuracy, limit of detection (LOD) and limit of quantification (LOQ) and sample stability.

Blank (n=6) and fortified (ABZ, ABZSO, ABZSO_2_ and IS) serum and urine samples were analyzed in order to demonstrate the capacity of the method to measure the analytes in presence of endogenous compounds (selectivity). The linearity was tested by constructing calibration curves for each compound in both matrixes. In serum the calibration ranges for ABZ, ABZSO and ABZSO_2_ were: 0.025-2 µg/mL using six different concentrations (n=3) (0.025, 0.05, 0.1, 0.5, 1 and 2 µg/mL), while in urine the range of calibration were for: ABZ and ABZSO_2_, 0.025-1 µg/mL, using five different concentrations (n=3) (0.025, 0.05, 0.1, 0.5, and 1 µg/mL), for ABZSO 0.025- 5 µg/mL, using seven different concentrations (n=3) (0.025, 0.05, 0.1, 0.5, 1, 3 and 5 µg/mL). The data were analysed for linearity using the least-squares regression method, using the Run Test and ANOVA to determine if the data differed from a straight line. Absolute recoveries for ABZ, ABZSO and ABZSO_2_ from human serum and urine were assessed at three different concentration levels (n=6). The extraction efficiency of the three molecules under study was determined by comparison of the detector responses (peak areas) obtained from fortified blank samples (serum and urine) with the peak areas resulting from direct injections of equivalent quantities of standard solutions in mobile phase. Accuracy was determined by evaluation of replicates (n=6) of drug-free serum or urine samples fortified with each compound (ABZ, ABZSO and ABZSO_2_) at three different concentrations of the calibration range. Accuracy of the method was measured by the differences between observed and calculated concentration results obtained in different days (inter-day at 3 consecutive working days) and expressed as the relative error (%RE). The intra and inter-day precision of the analytical method for either serum or urine was determined by the evaluation of replicates (n=6) of each analyte (ABZ, ABZSO and ABZSO_2_) at three different concentrations across three days of analysis. Precision was expressed as coefficient of variation (%CV). The theoretical limit of detection (LOD) was estimated integrating the baseline noise of the HPLC system in the area covering the mean retention time of each compound in six (n=6) blank (serum or urine) samples spiked with the IS and defined as the mean baseline noise/IS peak area ratio plus three standard deviations (SD). The limit of quantification (LOQ) was defined as the lowest drug concentration (n=6) in the serum or urine standard curve that could be quantified with a precision not exceeding 20% and accuracy within 20% of nominal. For stability assessment, blanks either of serum or urine samples were fortified (ABZ, ABZSO and ABZSO_2_) at two different concentrations (n=5) and stored at -18 ºC. Samples were assayed after 7 day-period. Additionally, freeze/thaw studies were conducted (3 freeze/thaw cycles – one cycle per day) at two different concentrations (n=5). Stability in human serum and urine was considered acceptable if the mean concentration obtained at the specified time point agrees with those of the freshly fortified control sample within ± 20%. Stability was determined as CV (%) between analysed samples.

**ANALYTICAL METHOD VALIDATION: RESULTS**

The work reported here describes the development and complete validation of a precise, reliable and simple reversed-phase HPLC method to quantify ABZ, ABZSO and ABZSO_2_ in serum and urine from humans. Under the described chromatographic conditions, the **mean retention times** were: **ABZSO: 4.3 min; ABZSO_2_: 6.7 min; ABZ: 11.7 min**. The total run time for the method was 16 min. The procedures employed for the extraction of the molecules were simple and highly efficient. No major endogenous chromatographic peaks, which could interfere with the resolution of drugs, were observed. The blank samples were free of interferences in the time regions of analytical interest.

**Standard calibration curves** for ABZ, ABZSO and ABZSO_2_ in serum and urine from humans were obtained using the linear least squares regression procedure. The area ratio values obtained after blank matrixes fortification to determined **linearity** are shown in **Tables: Aa**, **Ab** and **Ac** **(serum)**, and **Tables Ba, Bb** and **Bc (urine)**.

The **mean absolute recoveries** for ABZ, ABZSO and ABZSO_2_ in the different matrixes at three concentrations levels are shown in **Tables**: **Ca**, **Cb** and **Cc (serum)**, and **Tables** **Da**, **Db** and **Dc** **(urine)**. Recoveries from serum were very good (>70 %) for ABZSO ABZSO_2_ and ABZ, with mean values of 96.3, 92.1 and 75.5 %, respectively. In urine, recoveries for ABZSO and ABZSO_2_ were very good with mean values of 88.5 and 83.4%, respectively, being lower for ABZ (70.2%).

The ABZSO, ABZSO_2_ and ABZ chromatographic analyses of three drug concentration values were used to determine inter-day precision and inter-day accuracy. The **precision** results are shown in **Tables** **Ea**, **Eb** and **Ec** **(serum)**, and **Fa, Fb** and **Fc** **(urine)**. The **accuracy** results are shown in **Tables Ga**, **Gb** and **Gc (serum)**, and **Ha, Hb** and **Hc (urine)**. The method exhibited a high degree of inter-day precision for the three molecules demonstrated by CV always < 10% in both serum and urine. The accuracy was good, with mean RE values < 10% in all cases.

**Stability** assay were based in analysis of blank samples of each matrixes fortified with the three molecules at time 0 (n=3), and 7 (n=5) days post-freezing; and after 3 freeze/thaw cycles (n=5). The coefficients of variation after either storing at -18 ºC or freeze/thaw cycles variations were always < 15% for ABZSO, ABZSO_2_ and ABZ in both matrixes evaluated, indicating no significant degradation of molecules in these conditions. The stability results are shown in **Tables Ia**, **Ib** and **Ic (serum)**, and **Ja**, **Jb** and **Jc (urine).**

The **LOD** determined for ABZ, ABZSO and ABZSO_2_ in serum and urine are reported in **Tables Ka**, **Kb** and **Kc (serum)**, and **La**, **Lb** and **Lc (urine)**. The **LOQ** are shown in **Tables Ma**, **Mb** and **Mc (serum)**, **Na**, **Nb** and **Nc (urine)**.

The chromatograms corresponding to the **free drug** samples and ABZ, ABZSO and ABZSO_2_ **spiked** samples of either serum or urine from humans, are shown in **Fig S1** and **S2** (serum and urine, respectively).

**COMPLEMENTARY WORK ON THE CHARACTERIZATION OF THE URINARY METABOLITES PROFILE**

From the performed analytical and chromatographic work in urine samples from ABZ-treated volunteers, some complementary and useful information was obtained. In addition to the ABZ sulphoxide metabolite peak, other “relevant chromatographic peak” was observed from the analysis of the urinary samples. Some further analytical work was required to identify this “unknown chromatographic peak”, which was chemically identified as an oxidative amino-ABZ sulphone derivative. The mean retention time was **4.11** min under the previously described chromatographic conditions and it was detected between 4 and 72 h post-treatment. Although this metabolite was not included in the validation of the analytical methodology, its presence was checked by direct injections of known quantities of an amino-ABZSO_2_ reference standard (99% pure) solutions in mobile phase. The chromatograms corresponding to the amino-ABZSO_2_ metabolite spiked either in mobile phase samples or an experimental urine sample are shown in **Fig S3**.

Tables of the method validation to quantify ABZ, ABZSO and ABZSO_2_ in serum and urine from humans are presented below.

**Table Aa**

| **ABZSO concentration**  **(µg/mL)** | **ABZSO / OBZ (*)** |
| --- | --- |
|  | 0.025 |
| **0.025** | 0.024 |
|  | 0.023 |
|  | 0.050 |
| **0.05** | 0.049 |
|  | 0.051 |
|  | 0.094 |
| **0.1** | 0.094 |
|  | 0.093 |
|  | 0.471 |
| **0.5** | 0.459 |
|  | 0.441 |
|  | 0.933 |
| **1** | 0.914 |
|  | 0.903 |
|  | 1.832 |
| **2** | 1.815 |
|  | 1.876 |

(*): Peak area ratio obtained for ABZSO and oxibendazole (OBZ) internal standard in serum

**Table Ab**

| **ABZSO_2_ concentration**  **(µg/mL)** | **ABZSO_2_ / OBZ (*)** |
| --- | --- |
|  | 0.018 |
| **0.025** | 0.017 |
|  | 0.019 |
|  | 0.055 |
| **0.05** | 0.051 |
|  | 0.057 |
|  | 0.091 |
| **0.1** | 0.085 |
|  | 0.084 |
|  | 0.356 |
| **0.5** | 0.382 |
|  | 0.390 |
|  | 0.778 |
| **1** | 0.781 |
|  | 0.776 |
|  | 1.556 |
| **2** | 1.483 |
|  | 1.534 |

(*): Peak area ratio obtained for ABZSO_2_ and oxibendazole (OBZ) internal standard in plasma.

**Table Ac**

| **ABZ concentration**  **(µg/mL)** | **ABZ / OBZ (*)** |
| --- | --- |
|  | 0.013 |
| **0.025** | 0.012 |
|  | 0.011 |
|  | 0.022 |
| **0.05** | 0.023 |
|  | 0.024 |
|  | 0.044 |
| **0.1** | 0.045 |
|  | 0.043 |
|  | 0.209 |
| **0.5** | 0.221 |
|  | 0.218 |
|  | 0.411 |
| **1** | 0.399 |
|  | 0.409 |
|  | 0.798 |
| **2** | 0.833 |
|  | 0.838 |

(*): Peak area ratio obtained for ABZ and oxibendazole (OBZ) internal standard in plasma.

**Table Ba**

| **ABZSO concentration**  **(µg/mL)** | **ABZSO / OBZ (*)** |
| --- | --- |
|  | 0.052 |
| **0.025** | 0.058 |
|  | 0.060 |
|  | 0.112 |
| **0.05** | 0.107 |
|  | 0.107 |
|  | 0.223 |
| **0.1** | 0.230 |
|  | 0.243 |
|  | 1.044 |
| **0.5** | 1.039 |
|  | 1.046 |
|  | 1.945 |
| **1** | 1.830 |
|  | 1.918 |
|  | 5.470 |
| **3** | 5.445 |
|  | 5.340 |
|  | 9.024 |
| **5** | 8.779 |
|  | 8.976 |

(*): Peak area ratio obtained for ABZSO and oxibendazole (OBZ) internal standard in urine.

**Table Bb**

| **ABZSO_2_ concentration**  **(µg/mL)** | **ABZSO_2_ / OBZ (*)** |
| --- | --- |
|  | 0.041 |
| **0.025** | 0.044 |
|  | 0.040 |
|  | 0.092 |
| **0.05** | 0.094 |
|  | 0.090 |
|  | 0.168 |
| **0.1** | 0.177 |
|  | 0.168 |
|  | 0.883 |
| **0.5** | 0.842 |
|  | 0.804 |
|  | 1.687 |
| **1** | 1.659 |
|  | 1.656 |

(*): Peak area ratio obtained for ABZSO_2_ and oxibendazole (OBZ) internal standard in urine.

**Table Bc**

| **ABZ concentration**  **(µg/mL)** | **ABZ / OBZ (*)** |
| --- | --- |
|  | 0.031 |
| **0.025** | 0.029 |
|  | 0.028 |
|  | 0.046 |
| **0.05** | 0.048 |
|  | 0.045 |
|  | 0.093 |
| **0.1** | 0.090 |
|  | 0.096 |
|  | 0.438 |
| **0.5** | 0.436 |
|  | 0.461 |
|  | 0.876 |
| **1** | 0.944 |
|  | 0.951 |

(*): Peak area ratio obtained for ABZ and oxibendazole (OBZ) internal standard in urine.

**Table Ca**

| **Concentration**  **ABZSO (µg/mL)** | **PA**  **In methanol** | **PA**  **in serum** | **Recovery**  **(%)** |
| --- | --- | --- | --- |
| **0.05** | 25748 | 25174 | 97.8 |
|  | 24713 | 24288 | 98.3 |
|  | 26354 | 25912 | 98.3 |
|  | 25842 | 25516 | 98.7 |
|  | 25953 | 25686 | 99.0 |
|  | 25952 | 25686 | 99.0 |
| **Mean** |  |  | **98.5** |
| **0.5** | 245355 | 233313 | 95.1 |
|  | 248053 | 228551 | 92.1 |
|  | 265559 | 287567 | 108.3 |
|  | 252383 | 22437 | 88.9 |
|  | 246217 | 242361 | 98.4 |
|  | 258847 | 221093 | 85.4 |
| **Mean** |  |  | **94.7** |
| **1** | 448969 | 438685 | 97.7 |
|  | 517888 | 484601 | 93.6 |
|  | 478506 | 439207 | 91.8 |
|  | 468506 | 460270 | 98.2 |
|  | 484935 | 451561 | 93.1 |
|  | 528131 | 479021 | 90.7 |
| **Mean** |  |  | **95.9** |
| **Total mean** | | | **96.3** |

PA: Chromatographic peak area.

**Table Cb**

| **Concentration ABZSO_2_ (µg/mL)** | **PA**  **in methanol** | **PA**  **in serum** | **Recovery**  **(%)** |
| --- | --- | --- | --- |
| **0.05** | 28957 | 25748 | 88.9 |
|  | 27966 | 27008 | 96.6 |
|  | 28290 | 28134 | 99.4 |
|  | 25970 | 29762 | 114.6 |
|  | 27501 | 20498 | 74.5 |
|  | 26196 | 24605 | 93.9 |
| **Mean** |  |  | **94.7** |
| **0.5** | 219353 | 194103 | 88.5 |
|  | 228143 | 178053 | 78.0 |
|  | 221562 | 190337 | 85.9 |
|  | 233365 | 243365 | 104.3 |
|  | 243365 | 240296 | 98.7 |
|  | 240296 | 188802 | 78.6 |
| **Mean** |  |  | **89.0** |
| **1** | 407448 | 391114 | 96.0 |
|  | 434173 | 395363 | 91.1 |
|  | 446107 | 459931 | 103.1 |
|  | 453363 | 459931 | 101.4 |
|  | 436982 | 374892 | 85.8 |
|  | 435615 | 388523 | 89.2 |
| **Mean** |  |  | **92.6** |
| **Total mean** | | | **92.1** |

PA: Chromatographic peak area.

**Table Cc**

| **Concentration ABZ (µg/mL)** | **PA**  **In methanol** | **PA**  **in serum** | **Recovery**  **(%)** |
| --- | --- | --- | --- |
| **0.05** | 14442 | 10342 | 71.6 |
|  | 15893 | 12548 | 79.0 |
|  | 16505 | 11396 | 69.0 |
|  | 15643 | 11943 | 76.3 |
|  | 14614 | 12143 | 83.1 |
|  | 15356 | 11869 | 77.3 |
| **Mean** |  |  | **76.1** |
| **0.25** | 144730 | 113699 | 78.6 |
|  | 146709 | 102700 | 70.0 |
|  | 139080 | 103053 | 74.1 |
|  | 144339 | 105115 | 72.8 |
|  | 136382 | 110211 | 80.8 |
|  | 145758 | 106495 | 73.1 |
| **Mean** |  |  | **74.9** |
| **1** | 282470 | 197525 | 69.9 |
|  | 289178 | 201799 | 69.8 |
|  | 259138 | 198975 | 76.8 |
|  | 276728 | 202870 | 73.3 |
|  | 278943 | 220103 | 78.9 |
|  | 284251 | 229744 | 80.8 |
| **Mean** |  |  | **75.3** |
| **Total mean** | | | **75.5** |

PA: Chromatographic peak area.

**Table Da**

| **Concentration ABZSO**  **(µg/mL)** | **PA**  **In methanol** | **PA**  **urine** | **Recovery**  **(%)** |
| --- | --- | --- | --- |
| **0.05** | 73155 | 60937 | 83.3 |
|  | 73264 | 62126 | 84.8 |
|  | 72479 | 63008 | 86.9 |
|  | 78636 | 72411 | 92.1 |
|  | 72479 | 66009 | 91.1 |
|  | 78636 | 75799 | 96.4 |
| **Mean** |  |  | **89.1** |
| **0.5** | 755271 | 506285 | 67.0 |
|  | 744040 | 693366 | 93.2 |
|  | 750034 | 630260 | 84.0 |
|  | 821023 | 815229 | 99.3 |
|  | 817941 | 782278 | 95.6 |
|  | 832623 | 766444 | 92.1 |
| **Mean** |  |  | **88.5** |
| **1** | 1415642 | 1364864 | 96.4 |
|  | 1451847 | 1399315 | 96.4 |
|  | 1565500 | 1162790 | 74.3 |
|  | 1478985 | 1156755 | 78.2 |
|  | 1461779 | 1420105 | 97.1 |
|  | 1492358 | 1257132 | 84.2 |
| **Mean** |  |  | **87.8** |
| **Total mean** | | | **88.5** |

PA: Chromatographic peak area.

**Table Db**

| **Concentration ABZSO_2_ (µg/mL)** | **PA**  **in methanol** | **PA**  **in urine** | **Recovery**  **(%)** |
| --- | --- | --- | --- |
| **0.05** | 64302 | 70419 | 109.5 |
|  | 64519 | 61123 | 94.7 |
|  | 64635 | 63511 | 98.3 |
|  | 70289 | 65057 | 92.6 |
|  | 64519 | 51790 | 80.3 |
|  | 64635 | 63496 | 98.2 |
| **Mean** |  |  | **90.2** |
| **0.5** | 679555 | 642016 | 94.5 |
|  | 669008 | 530773 | 79.3 |
|  | 675041 | 479712 | 71.1 |
|  | 737470 | 432372 | 58.6 |
|  | 732296 | 510069 | 69.7 |
|  | 749341 | 578216 | 77.2 |
| **Mean** |  |  | **75.1** |
| **1** | 1248384 | 1153816 | 92.4 |
|  | 1288220 | 1187106 | 92.2 |
|  | 1385693 | 1266474 | 91.4 |
|  | 1320800 | 1028729 | 77.9 |
|  | 1285128 | 1283725 | 99.9 |
|  | 1315074 | 1045312 | 79.5 |
| **Mean** |  |  | **86.4** |
| **Total mean** | | | **83.9** |

PA: Chromatographic peak area.

**Table Dc**

| **Concentration ABZ (µg/mL)** | **PA**  **in methanol** | **PA**  **in urine** | **Recovery**  **(%)** |
| --- | --- | --- | --- |
| **0.05** | 38940 | 27905 | 71.7 |
|  | 38977 | 31377 | 80.5 |
|  | 42351 | 29894 | 70.6 |
|  | 42162 | 27905 | 66.2 |
|  | 38317 | 28225 | 73.7 |
|  | 42643 | 31025 | 72.8 |
| **Mean** |  |  | **72.6** |
| **0.5** | 412191 | 255753 | 62.0 |
|  | 405416 | 222436 | 54.9 |
|  | 413338 | 267772 | 64.8 |
|  | 447098 | 291580 | 65.2 |
|  | 465476 | 335056 | 72.0 |
|  | 472510 | 268239 | 56.8 |
| **Mean** |  |  | **68.0** |
| **1** | 787897 | 599638 | 76.1 |
|  | 813657 | 675869 | 83.1 |
|  | 876522 | 557930 | 63.7 |
|  | 835096 | 605194 | 72.5 |
|  | 808730 | 761570 | 94.2 |
|  | 828525 | 650196 | 78.5 |
| **Mean** |  |  | **70.1** |
| **Total mean** | | | **70.2** |

PA: Chromatographic peak area.

**Table Ea**

| **Concentration ABZSO**  **(µg/mL)** | **ABZSO/OBZ (*)** | **Mean** | **Precision (CV )** |
| --- | --- | --- | --- |
| **0.05** | 0.053 | 0.051 | **3.25** |
|  | 0.049 |  |  |
|  | 0.052 |  |  |
|  | 0.051 |  |  |
|  | 0.050 |  |  |
|  | 0.050 |  |  |
| **0.5** | 0.471 | 0.464 | **3.16** |
|  | 0.455 |  |  |
|  | 0.476 |  |  |
|  | 0.459 |  |  |
|  | 0.480 |  |  |
|  | 0.441 |  |  |
| **1** | 0.87 | 0.905 | **2.92** |
|  | 0.91 |  |  |
|  | 0.90 |  |  |
|  | 0.93 |  |  |
|  | 0.93 |  |  |
|  | 0,88 |  |  |

(*): Peak area ratio of ABZSO and oxibendazole (OBZ) internal standard in human serum. CV: Coefficient of variation (%).

**Table Eb**

| **Concentration ABZSO_2_**  **(µg/mL)** | **ABZSO_2_/OBZ (*)** | **Mean** | **Precision (CV )** |
| --- | --- | --- | --- |
| **0.05** | 0.055 | 0.054 | **8.46** |
|  | 0.051 |  |  |
|  | 0.057 |  |  |
|  | 0.060 |  |  |
|  | 0.047 |  |  |
|  | 0.057 |  |  |
| **0.5** | 0.356 | 0.384 | **4.73** |
|  | 0.382 |  |  |
|  | 0.390 |  |  |
|  | 0.406 |  |  |
|  | 0.398 |  |  |
|  | 0.374 |  |  |
| **1** | 0.78 | 0.778 | **0.69** |
|  | 0.78 |  |  |
|  | 0.78 |  |  |
|  | 0.78 |  |  |
|  | 0.77 |  |  |
|  | 0.79 |  |  |

(*): Peak area ratio of ABZSO_2_ and oxibendazole (OBZ) internal standard in human serum. CV: Coefficient of variation (%).

**Table Ec**

| **Concentration**  **ABZ**  **(µg/mL)** | **ABZ/OBZ (*)** | **Mean** | **Precision (CV )** |
| --- | --- | --- | --- |
| **0.05** | 0.022 | 0.023 | **2.54** |
|  | 0.024 |  |  |
|  | 0.023 |  |  |
|  | 0.024 |  |  |
|  | 0.023 |  |  |
|  | 0.023 |  |  |
| **0.5** | 0.208 | 0.214 | **2.34** |
|  | 0.221 |  |  |
|  | 0.211 |  |  |
|  | 0.209 |  |  |
|  | 0.218 |  |  |
|  | 0.215 |  |  |
| **1** | 0.39 | 0.405 | **2.29** |
|  | 0.40 |  |  |
|  | 0.41 |  |  |
|  | 0.41 |  |  |
|  | 0.42 |  |  |
|  | 0.42 |  |  |

(*): Peak area ratio of ABZ and oxibendazole (OBZ) internal standard in human serum. CV: Coefficient of variation (%).

**Table Fa**

| **Concentration ABZSO**  **(µg/mL)** | **ABZSO/OBZ (*)** | **Mean** | **Precision (CV )** |
| --- | --- | --- | --- |
| **0.05** | 0.100 | 0.106 | **3.88** |
|  | 0.105 |  |  |
|  | 0.107 |  |  |
|  | 0.102 |  |  |
|  | 0.112 |  |  |
|  | 0.107 |  |  |
| **0.5** | 1.044 | 1.096 | **5.91** |
|  | 1.100 |  |  |
|  | 1.153 |  |  |
|  | 1.046 |  |  |
|  | 1.039 |  |  |
|  | 1.192 |  |  |
| **1** | 1.99 | 1.938 | **3.06** |
|  | 1.95 |  |  |
|  | 1.98 |  |  |
|  | 1.95 |  |  |
|  | 1.83 |  |  |
|  | 1.92 |  |  |

(*): Peak area ratio of ABZSO and oxibendazole (OBZ) internal standard in human urine. CV: Coefficient of variation (%).

**Table Fb**

| **Concentration ABZSO_2_**  **(µg/mL)** | **ABZSO_2_/OBZ (*)** | **Mean** | **Precision (CV )** |
| --- | --- | --- | --- |
| **0.05** | 0.085 | 0.091 | **4.14** |
|  | 0.092 |  |  |
|  | 0.094 |  |  |
|  | 0.090 |  |  |
|  | 0.096 |  |  |
|  | 0.090 |  |  |
| **0.25** | 0.883 | 0.878 | **5.69** |
|  | 0.842 |  |  |
|  | 0.804 |  |  |
|  | 0.893 |  |  |
|  | 0.949 |  |  |
|  | 0.900 |  |  |
| **1** | 1.69 | 1.663 | **2.66** |
|  | 1.66 |  |  |
|  | 1.66 |  |  |
|  | 1.73 |  |  |
|  | 1.65 |  |  |
|  | 1.59 |  |  |

(*): Peak area ratio of ABZSO_2_ and oxibendazole (OBZ) internal standard in human urine. CV: Coefficient of variation (%).

**Table Fc**

| **Concentration**  **ABZ**  **(µg/mL)** | **ABZ/OBZ (*)** | **Mean** | **Precision (CV )** |
| --- | --- | --- | --- |
| **0.05** | 0.046 | 0.048 | **6.65** |
|  | 0.053 |  |  |
|  | 0.051 |  |  |
|  | 0.046 |  |  |
|  | 0.048 |  |  |
|  | 0.045 |  |  |
| **0.5** | 0.438 | 0.433 | **3.94** |
|  | 0.414 |  |  |
|  | 0.417 |  |  |
|  | 0.435 |  |  |
|  | 0.461 |  |  |
|  | 0.436 |  |  |
| **1** | 0.88 | 0.954 | **5.48** |
|  | 0.94 |  |  |
|  | 0.95 |  |  |
|  | 1.02 |  |  |
|  | 0.98 |  |  |
|  | 0.99 |  |  |

(*): Peak area ratio of ABZ and the oxibendazole (OBZ) internal standard in human urine. CV: Coefficient of variation (%).

**Table Ga**

| **Fortified ABZSO concentration (µg/mL)** | **Quantified concentration (µg/mL)** | **Accuracy^(1)^**  **(%)** |
| --- | --- | --- |
| **0.05** | 0.054 | 8.0 |
|  | 0.049 | -2.0 |
|  | 0.052 | 4.0 |
|  | 0.051 | 2.0 |
|  | 0.050 | 0.0 |
|  | 0.050 | 0.0 |
| **Mean** |  | **2.67** |
| **0.5** | 0.510 | 2.0 |
|  | 0.539 | 7.8 |
|  | 0.444 | -11.2 |
|  | 0.495 | -1.0 |
|  | 0.518 | 3.6 |
|  | 0.475 | -5.0 |
| **Mean** |  | **5.1** |
| **1** | 0.944 | -5.6 |
|  | 1.033 | 3.3 |
|  | 0.978 | -2.2 |
|  | 1.009 | 0.9 |
|  | 1.010 | 1.0 |
|  | 1.100 | 10.0 |
| **Mean** |  | **3.8** |

(1) Expressed as the relative error (%RE).

**Table Gb**

| **Fortified ABZSO_2_ concentration (µg/mL)** | **Quantified concentration (µg/mL)** | **Accuracy^(1)^**  **(%)** |
| --- | --- | --- |
| **0.05** | 0.049 | -2.0 |
|  | 0.044 | -12.0 |
|  | 0.052 | 4.0 |
|  | 0.056 | 12.0 |
|  | 0.039 | -22.0 |
|  | 0.052 | 4.0 |
| **Mean** |  | **4.40** |
| **0.5** | 0.449 | -10.2 |
|  | 0.484 | -3.2 |
|  | 0.494 | -1.2 |
|  | 0.516 | 3.2 |
|  | 0.505 | 1.0 |
|  | 0.473 | -5.4 |
| **Mean** |  | **4.03** |
| **1** | 1.010 | 1.0 |
|  | 1.010 | 1.0 |
|  | 1.000 | 0.0 |
|  | 1.000 | 0.0 |
|  | 1.010 | 1.0 |
|  | 1.020 | 2.0 |
| **Mean** |  | **0.83** |

(1) Expressed as the relative error (%RE).

**Table Gc**

| **Fortified ABZ concentration (µg/mL)** | **Quantified concentration (µg/mL)** | **Accuracy^(1)^**  **(%)** |
| --- | --- | --- |
| **0.05** | 0.045 | -10.0 |
|  | 0.050 | 0.0 |
|  | 0.048 | -4.0 |
|  | 0.050 | 0.0 |
|  | 0.048 | -4.0 |
|  | 0.048 | -4.0 |
| **Mean** |  | **3.67** |
| **0.25** | 0.499 | -0.2 |
|  | 0.531 | 6.2 |
|  | 0.506 | 1.2 |
|  | 0.502 | 0.4 |
|  | 0.524 | 4.8 |
|  | 0.509 | 1.8 |
| **Mean** |  | **2.56** |
| **1** | 0.951 | -4.9 |
|  | 0.965 | -3.5 |
|  | 0.990 | -1.0 |
|  | 0.995 | -0.5 |
|  | 0.953 | -4.7 |
|  | 0.980 | -2.0 |
| **Mean** |  | **2.92** |

(1) Expressed as the relative error (%RE).

**Table Ha**

| **Fortified ABZSO**  **concentration (µg/mL)** | **Quantified concentration (µg/mL)** | **Accuracy^(1)^**  **(%)** |
| --- | --- | --- |
| **0.05** | 0.042 | -16.0 |
|  | 0.045 | -10.0 |
|  | 0.046 | -8.0 |
|  | 0.043 | -14.0 |
|  | 0.048 | -4.0 |
|  | 0.046 | -8.0 |
| **Mean** |  | **10** |
| **0.5** | 0.480 | -4.0 |
|  | 0.500 | 0.0 |
|  | 0.530 | 6.0 |
|  | 0.480 | -4.0 |
|  | 0.481 | -3.8 |
|  | 0.550 | 10.0 |
| **Mean** |  | **4.63** |
| **1** | 1.080 | 8.0 |
|  | 1.060 | 6.0 |
|  | 1.070 | 7.0 |
|  | 1.050 | 5.0 |
|  | 0.990 | -1.0 |
|  | 1.040 | 4.0 |
| **Mean** |  | **5.17** |

(1) Expressed as the relative error (%RE).

**Table Hb**

| **Fortified ABZSO_2_ concentration (µg/mL)** | **Quantified concentration (µg/mL)** | **Accuracy^(1)^**  **(%)** |
| --- | --- | --- |
| **0.05** | 0.046 | -8.0 |
|  | 0.050 | 0.0 |
|  | 0.051 | 2.0 |
|  | 0.049 | -2.0 |
|  | 0.052 | 4.0 |
|  | 0.049 | -2.0 |
| **Mean** |  | **3.0** |
| **0.5** | 0.520 | 4.0 |
|  | 0.497 | -0.6 |
|  | 0.474 | -5.2 |
|  | 0.527 | 5.4 |
|  | 0.561 | 12.2 |
|  | 0.532 | 6.4 |
| **Mean** |  | **5.63** |
| **1** | 1.000 | 0.0 |
|  | 0.984 | -1.6 |
|  | 0.982 | -1.8 |
|  | 1.020 | 2.0 |
|  | 0.982 | -1.8 |
|  | 0.946 | -5.4 |
| **Mean** |  | **2.1** |

(1) Expressed as the relative error (%RE).

**Table Hc**

| **Fortified ABZ concentration (µg/mL)** | **Quantified concentration (µg/mL)** | **Accuracy^(1)^**  **(%)** |
| --- | --- | --- |
| **0.05** | 0.049 | -2.0 |
|  | 0.056 | 12.0 |
|  | 0.054 | 8.0 |
|  | 0.049 | -2.0 |
|  | 0.051 | 2.0 |
|  | 0.048 | -4.0 |
| **Mean** |  | **5.0** |
| **0.5** | 0.473 | -5.4 |
|  | 0.447 | -10.6 |
|  | 0.450 | -10.0 |
|  | 0.470 | -6.0 |
|  | 0.498 | -0.4 |
|  | 0.471 | -5.8 |
| **Mean** |  | **6.37** |
| **0.25** | 0.947 | -5.3 |
|  | 1.020 | 2.0 |
|  | 1.020 | 2.0 |
|  | 1.100 | 10.0 |
|  | 1.060 | 6.0 |
|  | 1.070 | 7.0 |
| **Mean** |  | **5.38** |

(1) Expressed as the relative error (%RE).

**Table Ia**

| **Stability factor** |  | **Concentration** | **Area ratio(*****)** | **CV** |
| --- | --- | --- | --- | --- |
|  |  | **(µg/mL)** |  |  |
| **Stored at -18°C** | **0 days** | **0.1** | 0.093 | **5.56** |
|  | **0 days** |  | 0.094 |  |
|  | **0 days** |  | 0.093 |  |
|  | **7 days** |  | 0.095 |  |
|  | **7 days** |  | 0.098 |  |
|  | **7 days** |  | 0.110 |  |
|  | **7 days** |  | 0.098 |  |
|  | **7days** |  | 0.097 |  |
| **Stored at -18°C** | **0 days** | **1** | 1.11 | **6.95** |
|  | **0 days** |  | 1.07 |  |
|  | **0 days** |  | 1.07 |  |
|  | **7 days** |  | 1.13 |  |
|  | **7 days** |  | 1.26 |  |
|  | **7 days** |  | 1.04 |  |
|  | **7 days** |  | 1.02 |  |
|  | **7 days** |  | 1.05 |  |
| **Freezed/thawed cycles** | **0** | **0.1** | 0.093 | **6.20** |
|  | **0** |  | 0.094 |  |
|  | **0** |  | 0.093 |  |
|  | **3** |  | 0.107 |  |
|  | **3** |  | 0.098 |  |
|  | **3** |  | 0.087 |  |
|  | **3** |  | 0.095 |  |
|  | **3** |  | 0.092 |  |
| **Freezed/thawed cycles** | **0** | **1** | 0.90 | **1.72** |
|  | **0** |  | 0.93 |  |
|  | **0** |  | 0.93 |  |
|  | **3** |  | 0.91 |  |
|  | **3** |  | 0.89 |  |
|  | **3** |  | 0.93 |  |
|  | **3** |  | 0.94 |  |
|  | **3** |  | 0.92 |  |

(*): Peak area ratio of ABZSO and the internal standard used in serum.

**Table Ib**

| **Stability**  **factor** |  | **Concentration** | **Area ratio(*****)** | **CV** |
| --- | --- | --- | --- | --- |
|  |  | **(µg/mL)** |  |  |
| **Stored at -18°C** | **0 days** | **0.1** | 0.091 | **6.8** |
|  | **0 days** |  | 0.085 |  |
|  | **0 days** |  | 0.084 |  |
|  | **7 days** |  | 0.093 |  |
|  | **7 days** |  | 0.088 |  |
|  | **7 days** |  | 0.079 |  |
|  | **7 days** |  | 0.082 |  |
|  | **7 days** |  | 0.078 |  |
| **Stored at -18°C** | **0 days** | **1** | 0.78 | **6.7** |
|  | **0 days** |  | 0.78 |  |
|  | **0 days** |  | 0.78 |  |
|  | **7 days** |  | 0.79 |  |
|  | **7 days** |  | 0.67 |  |
|  | **7 days** |  | 0.82 |  |
|  | **7 days** |  | 0.84 |  |
|  | **7 days** |  | 0.83 |  |
| **Freezed/thawed cycles** | **0** | **0.1** | 0.091 | **8.62** |
|  | **0** |  | 0.085 |  |
|  | **0** |  | 0.084 |  |
|  | **3** |  | 0.070 |  |
|  | **3** |  | 0.077 |  |
|  | **3** |  | 0.091 |  |
|  | **3** |  | 0.081 |  |
|  | **3** |  | 0.089 |  |
| **Freezed/thawed cycles** | **0** | **1** | 0.78 | **0.87** |
|  | **0** |  | 0.78 |  |
|  | **0** |  | 0.78 |  |
|  | **3** |  | 0.77 |  |
|  | **3** |  | 0.76 |  |
|  | **3** |  | 0.78 |  |
|  | **3** |  | 0.77 |  |
|  | **3** |  | 0.78 |  |

(*): Peak area ratio of ABZSO_2_ and the internal standard used in serum.

**Table Ic**

| **Stability**  **factor** |  | **Concentration** | **Area ratio(*****)** | **CV** |
| --- | --- | --- | --- | --- |
|  |  | **(µg/mL)** |  |  |
| **Stored at -18°C** | **0 days** | **0.1** | 0.046 | **2.39** |
|  | **0 days** |  | 0.045 |  |
|  | **0 days** |  | 0.047 |  |
|  | **7 days** |  | 0.046 |  |
|  | **7 days** |  | 0.044 |  |
|  | **7 days** |  | 0.045 |  |
|  | **7 days** |  | 0.044 |  |
|  | **7 days** |  | 0.046 |  |
| **Stored at -18°C** | **0 days** | **1** | 0.409 | **5.45** |
|  | **0 days** |  | 0.411 |  |
|  | **0 days** |  | 0.415 |  |
|  | **7 days** |  | 0.427 |  |
|  | **7 days** |  | 0.366 |  |
|  | **7 days** |  | 0.410 |  |
|  | **7 days** |  | 0.438 |  |
|  | **7 days** |  | 0.435 |  |
| **Freezed/thawed cycles** | **0** | **0.1** | 0.046 | **5.58** |
|  | **0** |  | 0.045 |  |
|  | **0** |  | 0.047 |  |
|  | **3** |  | 0.049 |  |
|  | **3** |  | 0.046 |  |
|  | **3** |  | 0.042 |  |
|  | **3** |  | 0.043 |  |
|  | **3** |  | 0.042 |  |
| **Freezed/thawed cycles** | **0** | **1** | 0.41 | **2.47** |
|  | **0** |  | 0.41 |  |
|  | **0** |  | 0.42 |  |
|  | **3** |  | 0.39 |  |
|  | **3** |  | 0.42 |  |
|  | **3** |  | 0.42 |  |
|  | **3** |  | 0.43 |  |
|  | **3** |  | 0.42 |  |

(*): Peak area ratio of ABZ and the internal standard used in serum.

**Table Ja**

| **Stability**  **Factor** |  | **Concentration** | **Area ratio(*****)** | **CV** |
| --- | --- | --- | --- | --- |
|  |  | **(µg/mL)** |  |  |
| **Stored at -18°C** | **0 days** | **01** | 0.260 | **9.96** |
|  | **0 days** |  | 0.243 |  |
|  | **0 days** |  | 0.221 |  |
|  | **7 days** |  | 0.207 |  |
|  | **7 days** |  | 0.212 |  |
|  | **7 days** |  | 0.197 |  |
|  | **7 days** |  | 0.200 |  |
|  | **7 days** |  | 0.210 |  |
| **Stored at -18°C** | **0 days** | **0.5** | 0.91 | **6.23** |
|  | **0 days** |  | 0.87 |  |
|  | **0 days** |  | 0.96 |  |
|  | **7 days** |  | 0.91 |  |
|  | **7 days** |  | 0.82 |  |
|  | **7 days** |  | 0.89 |  |
|  | **7 days** |  | 1.01 |  |
|  | **7 days** |  | 0.92 |  |
| **Freezed/thawed cycles** | **0** | **0.25** | 0.260 | **7.41** |
|  | **0** |  | 0.243 |  |
|  | **0** |  | 0.221 |  |
|  | **3** |  | 0.203 |  |
|  | **3** |  | 0.229 |  |
|  | **3** |  | 0.225 |  |
|  | **3** |  | 0.221 |  |
|  | **3** |  | 0.221 |  |
| **Freezed/thawed cycles** | **0** | **1** | 1.10 | **3.86** |
|  | **0** |  | 1.15 |  |
|  | **0** |  | 1.05 |  |
|  | **3** |  | 1.12 |  |
|  | **3** |  | 1.15 |  |
|  | **3** |  | 1.11 |  |
|  | **3** |  | 1.07 |  |
|  | **3** |  | 1.04 |  |

(*): Peak area ratio of ABZSO and the internal standard used urine.

**Table Jb**

| **Stability**  **Factor** |  | **Concentration** | **Area ratio(*****)** | **CV** |
| --- | --- | --- | --- | --- |
|  |  | **(µg/mL)** |  |  |
| **Stored at -18°C** | **0 days** | **0.1** | 0.168 | **7.1** |
|  | **0 days** |  | 0.182 |  |
|  | **0 days** |  | 0.183 |  |
|  | **7 days** |  | 0.207 |  |
|  | **7 days** |  | 0.202 |  |
|  | **7 days** |  | 0.175 |  |
|  | **30 days** |  | 0.182 |  |
|  | **30 days** |  | 0.191 |  |
| **Stored at -18°C** | **0 days** | **0.5** | 0.91 | **11.9** |
|  | **0 days** |  | 0.87 |  |
|  | **0 days** |  | 0.96 |  |
|  | **7 days** |  | 1.12 |  |
|  | **7 days** |  | 1.13 |  |
|  | **7 days** |  | 1.14 |  |
|  | **30 days** |  | 1.17 |  |
|  | **30 days** |  | 1.18 |  |
| **Freezed/thawed cycles** | **0** | **0.1** | 0.168 | **14.6** |
|  | **0** |  | 0.182 |  |
|  | **0** |  | 0.183 |  |
|  | **3** |  | 0.153 |  |
|  | **3** |  | 0.118 |  |
|  | **3** |  | 0.139 |  |
|  | **3** |  | 0.151 |  |
|  | **3** |  | 0.140 |  |
| **Freezed/thawed cycles** | **0** | **0.5** | 0.89 | **12.1** |
|  | **0** |  | 0.95 |  |
|  | **0** |  | 0.90 |  |
|  | **3** |  | 0.95 |  |
|  | **3** |  | 1.01 |  |
|  | **3** |  | 0.84 |  |
|  | **3** |  | 0.80 |  |
|  | **3** |  | 0.67 |  |

(*): Peak area ratio of ABZSO_2_ and the internal standard used in urine.

**Table Jc**

| **Stability**  **Factor** |  | **Concentration** | **Area ratio(*****)** | **CV** |
| --- | --- | --- | --- | --- |
|  |  | **(µg/mL)** |  |  |
| **Stored at -18°C** | **0 days** | **0.1** | 0.100 | **7.4** |
|  | **0 days** |  | 0.096 |  |
|  | **0 days** |  | 0.109 |  |
|  | **7 days** |  | 0.092 |  |
|  | **7 days** |  | 0.089 |  |
|  | **7 days** |  | 0.088 |  |
|  | **7 days** |  | 0.090 |  |
|  | **7 days** |  | 0.097 |  |
| **Stored at -18°C** | **0 days** | **0.5** | 0.435 | **7.7** |
|  | **0 days** |  | 0.461 |  |
|  | **0 days** |  | 0.436 |  |
|  | **7 days** |  | 0.474 |  |
|  | **7 days** |  | 0.514 |  |
|  | **7 days** |  | 0.533 |  |
|  | **7 days** |  | 0.455 |  |
|  | **7days** |  | 0.504 |  |
| **Freezed/thawed cycles** | **0** | **0.1** | 0.100 | **12.1** |
|  | **0** |  | 0.096 |  |
|  | **0** |  | 0.109 |  |
|  | **3** |  | 0.088 |  |
|  | **3** |  | 0.073 |  |
|  | **3** |  | 0.088 |  |
|  | **3** |  | 0.088 |  |
|  | **3** |  | 0.083 |  |
| **Freezed/thawed cycles** | **0** | **0.5** | 0.43 | **13.5** |
|  | **0** |  | 0.46 |  |
|  | **0** |  | 0.44 |  |
|  | **3** |  | 0.53 |  |
|  | **3** |  | 0.62 |  |
|  | **3** |  | 0.53 |  |
|  | **3** |  | 0.50 |  |
|  | **3** |  | 0.43 |  |

(*): Peak area ratio of ABZ and the internal standard used in urine.

**Table Ka**

| **Blank serum sample** | **Area ratio of the noise baseline (noise/IS) at the ABZSO retention time** |
| --- | --- |
| **1** | 0.006 |
| **2** | 0.008 |
| **3** | 0.007 |
| **4** | 0.003 |
| **5** | 0.004 |
| **6** | 0.001 |
| **Mean** | **0.005** |
| **SD** | **0.003** |
| **Mean+3SD** | **0.013** |
| **Limit of detection**  **(µg/mL)** | **0.013** |

SD: standard deviation

**Table Kb**

| **Blank serum sample** | **Area ratio of the noise baseline (noise/IS) at the ABZSO_2_** **retention time** |
| --- | --- |
| **1** | 0.002 |
| **2** | 0.002 |
| **3** | 0.002 |
| **4** | 0.001 |
| **5** | 0.002 |
| **6** | 0.002 |
| **Mean** | **0.002** |
| **SD** | **0.001** |
| **Mean+3SD** | **0.003** |
| **Limit of detection**  **(µg/mL)** | **0.001** |

SD: standard deviation

**Table Kc**

| **Blank serum sample** | **Area ratio of the noise baseline (noise/IS) at the ABZ** **retention time** |
| --- | --- |
| **1** | 0.0011 |
| **2** | 0.0013 |
| **3** | 0.0010 |
| **4** | 0.0051 |
| **5** | 0.0027 |
| **6** | 0.0030 |
| **Mean** | **0.0024** |
| **SD** | **0.0016** |
| **Mean+3SD** | **0.007** |
| **Limit of detection**  **(µg/mL)** | **0.009** |

SD: standard deviation

**Table La**

| **Blank urine sample** | **Area ratio of the noise baseline (noise/IS) at the ABZSO retention time** |
| --- | --- |
| **1** | 0.002 |
| **2** | 0.002 |
| **3** | 0.002 |
| **4** | 0.001 |
| **5** | 0.002 |
| **6** | 0.001 |
| **7** | 0.003 |
| **8** | 0.003 |
| **9** | 0.002 |
| **Mean** | **0.002** |
| **SD** | **0.001** |
| **Mean+3SD** | **0.004** |
| **Limit of detection**  **(µg/mL)** | **0.001** |

SD: standard deviation

**Table Lb**

| **Blank urine sample** | **Area ratio of the noise baseline (noise/IS) at the ABZSO_2_** **retention time** |
| --- | --- |
| **1** | 0.001 |
| **2** | 0.001 |
| **3** | 0.001 |
| **4** | 0.001 |
| **5** | 0.001 |
| **6** | 0.001 |
| **7** | 0.001 |
| **8** | 0.001 |
| **9** | 0.001 |
| **Mean** | **0.001** |
| **SD** | **0.0002** |
| **Mean+3SD** | **0.0017** |
| **Limit of detection**  **(µg/mL)** | **0.002** |

SD: standard deviation

**Table Lc**

| **Blank urine sample** | **Area ratio of the noise baseline (noise/IS) at the ABZ** **retention time** |
| --- | --- |
| **1** | 0.035 |
| **2** | 0.034 |
| **3** | 0.028 |
| **4** | 0.038 |
| **5** | 0.039 |
| **6** | 0.027 |
| **7** | 0.033 |
| **8** | 0.035 |
| **9** | 0.027 |
| **Mean** | **0.033** |
| **SD** | **0.005** |
| **Mean+3SD** | **0.047** |
| **Limit of detection**  **(µg/mL)** | **0.050** |

SD: standard deviation

**Table Ma**

| **LOQ RECOVERY** | | | |
| --- | --- | --- | --- |
| **ABZSO concentration (µg/mL)** | **Mobile phase PA** | **Serum PA** | **Recovery (%)** |
| **0.025** | 12984 | 12738 | 98.1 |
|  | 12657 | 12464 | 98.5 |
|  | 12192 | 12153 | 99.7 |
|  | 11877 | 11346 | 95.5 |
|  | 12192 | 11490 | 94.2 |
|  | 12877 | 11892 | 92.4 |
| **Mean** |  |  | **96.4** |

PA: Peak area.

| **LOQ ACCURACY** | | |
| --- | --- | --- |
| **Fortified ABZSO concentration (µg/mL)** | **Concentration quantified (µg/mL)** | **Accuracy^(1)^**  **(%)** |
| **0.025** | 0.023 | -8.0 |
|  | 0.020 | -20.0 |
|  | 0.021 | -16.0 |
|  | 0.021 | -16.0 |
|  | 0.022 | -12.0 |
|  | 0.023 | -8.0 |
| **Mean** |  | **10.33** |

1. Expressed as the relative error (%RE).

| **LOQ PRECISION** | |
| --- | --- |
| **ABZSO concentration (µg/mL)** | **ABZSO / IS (*)** |
| **0.025** | 0.021 |
|  | 0.022 |
|  | 0.021 |
|  | 0.020 |
|  | 0.021 |
|  | 0.020 |
| **Mean** | **0.021** |
| **CV (%)** | **3.78** |

(*): Peak area ratio of ABZSO and the internal standard (IS) used in human serum. CV: Coefficient of variation (%).

**Table Mb**

| **LOQ RECOVERY** | | | |
| --- | --- | --- | --- |
| **ABZSO_2_ concentration (µg/mL)** | **Mobile phase PA** | **Serum PA** | **Recovery (%)** |
| **0.05** | 28957 | 25748 | 88.9 |
|  | 27966 | 27008 | 96.6 |
|  | 28290 | 28134 | 99.4 |
|  | 25970 | 29762 | 114.6 |
|  | 27501 | 20498 | 74.5 |
|  | 26196 | 24605 | 93.9 |
| **Mean** |  |  | **94.7** |

PA: Peak area.

| **LOQ ACCURACY** | | |
| --- | --- | --- |
| **Fortified ABZSO_2_ concentration (µg/mL)** | **Concentration quantified (µg/mL)** | **Accuracy^(1)^**  **(%)** |
| **0.05** | 0.049 | -2.0 |
|  | 0.044 | -12.0 |
|  | 0.052 | 4.0 |
|  | 0.056 | 12.0 |
|  | 0.039 | -22.0 |
|  | 0.052 | 4.0 |
| **Mean** |  | **9.3** |

(1)Expressed as the relative error (%RE).

| **LOQ PRECISION** | |
| --- | --- |
| **ABZSO_2_ concentration (µg/mL)** | **ABZSO_2_ / IS (*)** |
| **0.05** | 0.055 |
|  | 0.051 |
|  | 0.057 |
|  | 0.060 |
|  | 0.047 |
|  | 0.057 |
| **Mean** | **0.054** |
| **CV (%)** | **8.46** |

(*): Peak area ratio of ABZSO_2_ and the internal standard (IS) used in human serum. CV: Coefficient of variation (%).

**Table Mc**

| **LOQ RECOVERY** | | | |
| --- | --- | --- | --- |
| **ABZ concentration (µg/mL)** | **Mobile phase PA** | **Serum PA** | **Recovery (%)** |
| **0.025** | 8057 | 5868 | 72.8 |
|  | 7517 | 5416 | 72.1 |
|  | 7312 | 5961 | 81.5 |
|  | 8113 | 6264 | 77.2 |
|  | 8112 | 6055 | 74.6 |
|  | 7822 | 5913 | 75.6 |
| **Mean** |  |  | **75.6** |

PA: Peak area.

| **LOQ ACCURACY** | | |
| --- | --- | --- |
| **Fortified ABZ concentration (µg/mL)** | **Concentration quantified (µg/mL)** | **Accuracy^(1)^**  **(%)** |
| **0.025** | 0.023 | -8.0 |
|  | 0.021 | -16.0 |
|  | 0.018 | -28.0 |
|  | 0.018 | -28.0 |
|  | 0.021 | -16.0 |
|  | 0.021 | -16.0 |
| **Mean** |  | **18.6** |

(1)Expressed as the relative error (%RE).

| **LOQ PRECISION** | |
| --- | --- |
| **ABZ concentration (µg/mL)** | **ABZ / IS (*)** |
| **0.025** | 0.013 |
|  | 0.012 |
|  | 0.011 |
|  | 0.011 |
|  | 0.012 |
|  | 0.012 |
| **Mean** | **0.012** |
| **CV (%)** | **4.72** |

(*): Peak area ratio of ABZ and the internal standard (IS) used in human serum. CV: Coefficient of variation (%).

**Table Na**

| **LOQ RECOVERY** | | | |
| --- | --- | --- | --- |
| **ABZSO concentration (µg/mL)** | **Mobile phase PA** | **Urine PA** | **Recovery (%)** |
| **0.025** | 36140 | 30340 | 84.0 |
|  | 39342 | 30572 | 77.7 |
|  | 37474 | 30254 | 80.7 |
|  | 34057 | 31836 | 93.5 |
|  | 34968 | 33585 | 96.0 |
|  | 37857 | 30548 | 80.7 |
| **Mean** |  |  | **85.4** |

PA: Peak area.

| **LOQ ACCURACY** | | |
| --- | --- | --- |
| **Fortified ABZSO concentration (µg/mL)** | **Concentration quantified (µg/mL)** | **Accuracy^(1)^**  **(%)** |
| **0.025** | 0.018 | -28.0 |
|  | 0.019 | -24.0 |
|  | 0.023 | -8.0 |
|  | 0.020 | -20.0 |
|  | 0.024 | -4.0 |
|  | 0.020 | -20.0 |
| **Mean** |  | **17.3** |

(1)Expressed as the relative error (%RE).

| **LOQ PRECISION** | |
| --- | --- |
| **ABZSO concentration (µg/mL)** | **ABZSO / IS (*)** |
| **0.025** | 0.048 |
|  | 0.049 |
|  | 0.058 |
|  | 0.052 |
|  | 0.060 |
|  | 0.052 |
| **Mean** | **0.053** |
| **CV (%)** | **9.7** |

(*): Peak area ratio of ABZSO and the internal standard (IS) used in human urine. CV: Coefficient of variation (%).

**Table Nb**

| **LOQ RECOVERY** | | | |
| --- | --- | --- | --- |
| **ABZSO_2_ concentration (µg/mL)** | **Mobile phase PA** | **Urine PA** | **Recovery (%)** |
| **0.025** | 31238 | 28753 | 92.0 |
|  | 37155 | 31614 | 85.1 |
|  | 33437 | 31915 | 95.4 |
|  | 31603 | 27915 | 88.3 |
|  | 31200 | 26153 | 83.8 |
|  | 33265 | 29470 | 88.6 |
| **Mean** |  |  | **88.9** |

PA: Peak area.

| **LOQ ACCURACY** | | |
| --- | --- | --- |
| **Fortified ABZSO_2_ concentration (µg/mL)** | **Concentration quantified (µg/mL)** | **Accuracy^(1)^**  **(%)** |
| **0.025** | 0.020 | -20.0 |
|  | 0.021 | -16.0 |
|  | 0.019 | -24.0 |
|  | 0.021 | -16.0 |
|  | 0.021 | -16.0 |
|  | 0.020 | -20.0 |
| **Mean** |  | **18.6** |

(1)Expressed as the relative error (%RE).

| **LOQ PRECISION** | |
| --- | --- |
| **ABZSO_2_ concentration (µg/mL)** | **ABZSO_2_ / IS (*)** |
| **0.025** | 0.041 |
|  | 0.044 |
|  | 0.040 |
|  | 0.044 |
|  | 0.044 |
|  | 0.042 |
| **Mean** | **0.042** |
| **CV (%)** | **3.99** |

(*): Peak area ratio of ABZSO_2_ and the internal standard (IS) used in human urine. CV: Coefficient of variation (%).

**Table Nc**

| **LOQ RECOVERY** | | | |
| --- | --- | --- | --- |
| **ABZ concentration (µg/mL)** | **Mobile phase PA** | **Urine PA** | **Recovery (%)** |
| **0.05** | 38940 | 27905 | 71.7 |
|  | 38977 | 31377 | 80.5 |
|  | 42351 | 29894 | 70.6 |
|  | 42162 | 27905 | 66.2 |
|  | 42158 | 28225 | 67.0 |
|  | 41211 | 31025 | 75.3 |
| **Mean** |  |  | **71.9** |

PA: Peak area.

| **LOQ ACCURACY** | | |
| --- | --- | --- |
| **Fortified ABZ concentration (µg/mL)** | **Concentration quantified (µg/mL)** | **Accuracy^(1)^**  **(%)** |
| **0.05** | 0.049 | -2.0 |
|  | 0.056 | 12.0 |
|  | 0.054 | 8.0 |
|  | 0.049 | -2.0 |
|  | 0.051 | 2.0 |
|  | 0.048 | -4.0 |
| **Mean** |  | **5** |

(1)Expressed as the relative error (%RE).

| **LOQ PRECISION** | |
| --- | --- |
| **ABZ concentration (µg/mL)** | **ABZ / IS (*)** |
| **0.05** | 0.046 |
|  | 0.053 |
|  | 0.051 |
|  | 0.046 |
|  | 0.048 |
|  | 0.045 |
| **Mean** | **0.048** |
| **CV (%)** | **6.65** |

(*): Peak area ratio of ABZ and the internal standard (IS) used in human urine. CV: Coefficient of variation (%).
