# Supplementary figures and images for: Assessment of serum pharmacokinetics and urinary excretion of albendazole and its metabolites in human volunteers
Source: PLoS Negl Trop Dis. 2018 Jan 18;12(1):e0005945. doi: 10.1371/journal.pntd.0005945 (PMC5773000; doi:10.1371/journal.pntd.0005945)

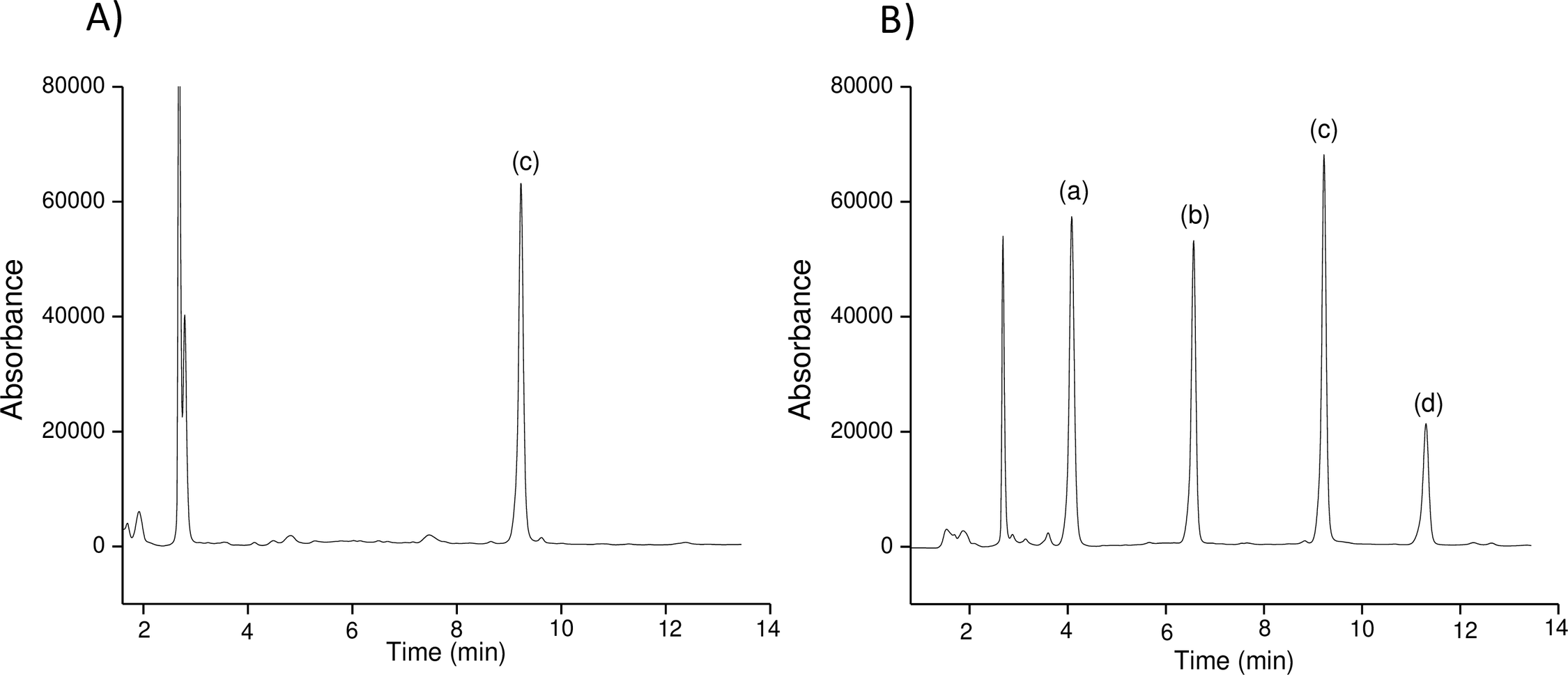

Supplement: S1 Fig — Chromatograms obtained from (A) drug-free serum sample spiked with oxibendazole (c) (as internal standard); (B) Serum sample spiked with albendazole sulphoxide; (a) (4.3 min), albendazole sulphone (b) (6.7 min), oxibendazole (c) and albendazole (d) (11.7 min). (TIF) [file pntd.0005945.s002.tif]

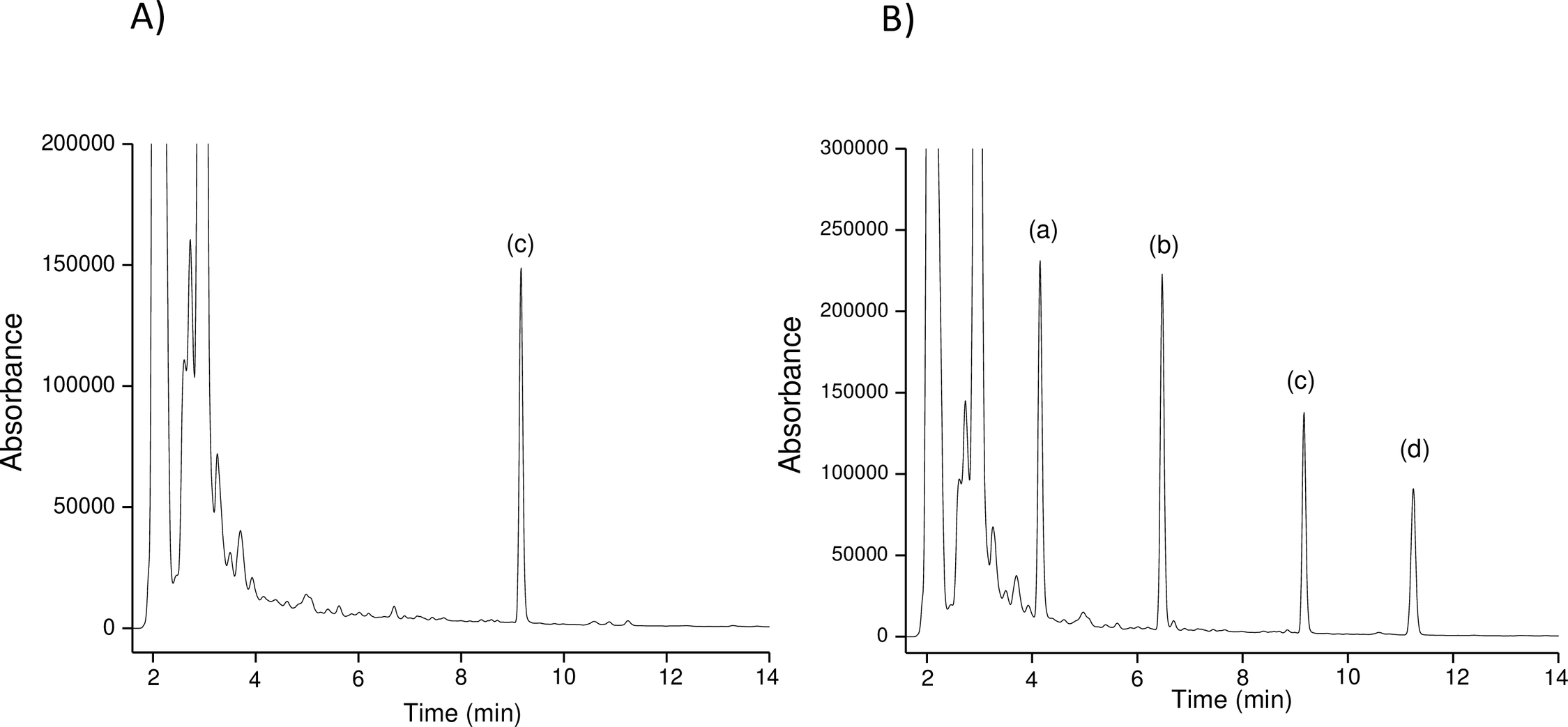

Supplement: S2 Fig — Chromatograms obtained from (A) drug-free urine sample spiked with oxibendazole (c) (as internal standard); (B) Urine sample spiked with albendazole sulphoxide; (a) (4.3 min), albendazole sulphone (b) (6.7 min), oxibendazole (c) and albendazole (d) (11.7 min). (TIF) [file pntd.0005945.s003.tif]

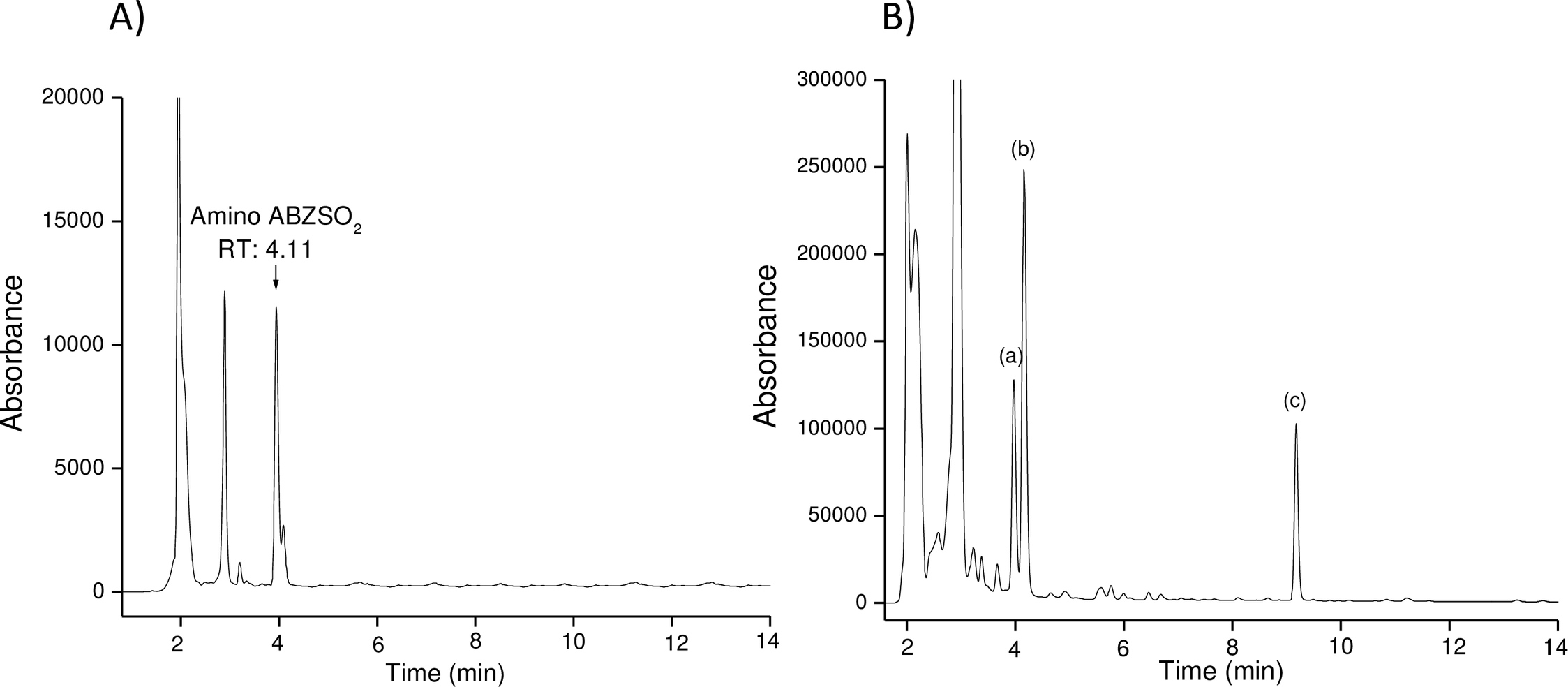

Supplement: S3 Fig — Shows the chromatograms corresponding to the amino-ABZSO2 metabolite spiked in mobile phase samples (A), and to an experimental urine sample (B), albendazole amino sulphone (a) (4.11 min); albendazole sulphoxide(b) (4.3 min); oxibendazole (c) (6.7 min). (TIF) [file pntd.0005945.s004.tif]
